# The rise of *Cynometra* (Leguminosae) and the fall of *Maniltoa*: a generic re-circumscription and the addition of 4 new species

**DOI:** 10.3897/phytokeys.127.29817

**Published:** 2019-07-19

**Authors:** Aleksandar Radosavljevic

**Affiliations:** 1 Chicago Botanic Garden, 1000 Lake Cook Road, Glencoe, Illinois 60022, USA Chicago Botanic Garden Glencoe United States of America; 2 National Museum of Natural History, Smithsonian Institution, 10th St and Constitution Ave NW, Washington, D. C. 20560, USA National Museum of Natural History, Smithsonian Institution Washington United States of America; 3 Northwestern University, 633 Clark St, Evanston, Illinois 60202, USA Northwestern University Evanston United States of America

**Keywords:** *Cynometra* L, Detaroideae, Leguminosae, *Maniltoa* Scheff, new species, taxonomy

## Abstract

*Cynometra* L. is a genus of ca. 85 species of shrubs to large trees. It is amongst the largest genera in the legume subfamily Detarioideae and one of the few with a pantropical distribution. Perhaps due to this wide distribution and high diversity, systematists and taxonomists have struggled with the classification of *Cynometra* and its close ally, the genus *Maniltoa* Scheff. Recent phylogenetic studies have shown that many of the African species are more closely related to other genera and that the genus *Maniltoa* is nested within a clade of Indo-Pacific *Cynometra*. Here, I present an emended circumscription of *Cynometra* that excludes the African species defined by jointed pedicels and dehiscent fruits and includes the species formerly recognised in *Maniltoa*. New combinations in *Cynometra* are also provided for those species that require them. Additionally, four new species of Neotropical *Cynometra* are described and illustrated: *Cynometracerebriformis***sp. nov.** from the lower Rio Trombetas in Brazil; *Cynometradwyerii***sp. nov.** from the Darien gap region of Panama; *C.tumbesiana***sp. nov.** from the dry tropical forests of Ecuador and Peru; and *C.steyermarkii***sp. nov.** from the foothills of the western Cordillera de la Costa in Venezuela.

## Introduction

**Figure 1.**
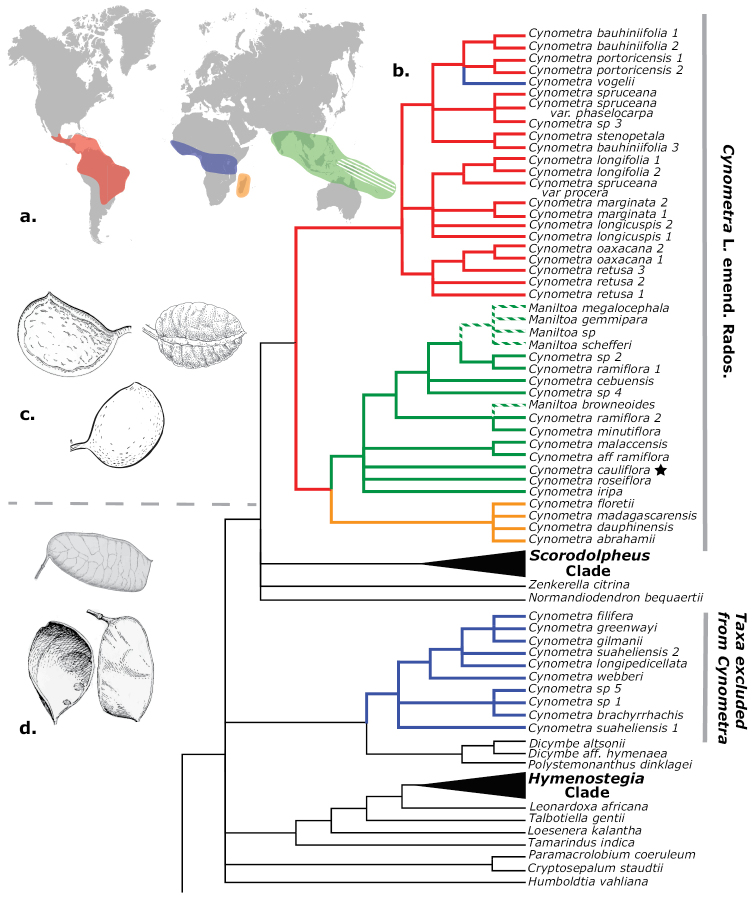
**A** Map showing global distribution of *Cynometra* and *Maniltoa* (white hatching) **B** ML majority rules consensus tree from Radosavljevic et al. (2017) with branches coloured by species distributions. Star indicates type species of *Cynometra***C** indehiscent fruits typical of *Cynometra* as circumscribed here; **D** dehiscent fruits typical of those species formerly included in *Cynometra*, but excluded by the new circumscription.

The genus *Cynometra* L. (Leguminosae) has a broad tropical distribution, is relatively species rich and many of its species are poorly represented in herbaria. The approximately 85 species of trees and (some) shrubs in the genus are spread somewhat evenly amongst four regions (Figure 1): the Neotropics (from southern Mexico and the Caribbean to northern Argentina; Dwyer 1958, Sprada Tavares and da Silva 1992), mainland tropical Africa (equatorial forest belt; Léonard 1951), Madagascar and the Comoros Islands (Du Puy et al. 2002) and the Indo-Pacific (extending from the Western Ghats eastward to Fiji; Knaap-van Meeuwen 1970, Smith 1985). Perhaps unsurprisingly, the genus has long troubled taxonomists and systematists who have struggled with its diversity in several attempts to revise and classify regional groupings of the species (Léonard 1951, Dwyer 1958, Knaap-van Meeuwen 1970). Further, its relationship to the much smaller Pacific genus *Maniltoa* Scheff. has remained equivocal. Several important phylogenetic studies of the Caesalpinioideae sensu lato suggested that *Cynometra* was not monophyletic (Bruneau et al. 2001, 2008). More recent studies, with greater taxonomic sampling, have provided additional evidence that the genus is polyphyletic and, furthermore, that one of the lineages is paraphyletic with respect to *Maniltoa* (Mackinder et al. 2013, Radosavljevic et al. 2017, de la Estrella et al. 2018). In order for the classification of *Cynometra* to reflect a monophyletic taxon, a new genus circumscription must be provided and *Maniltoa* should be subsumed into *Cynometra*. In addition, during the course of herbarium study and fieldwork, several undescribed species were revealed. Those species are here described. This paper is divided into three parts: I. A new circumscription of the genus *Cynometra*; II. The transfer of all species of *Maniltoa* into *Cynometra*; and III. Description of four new species of *Cynometra*.

## Materials and methods

The following herbaria allowed the author to study their collections in person or provided loans: A, ATH, BM, BRI, CANB, F, IAN, INPA, K, MG, MO, NY, P, RB, SING and US. Morphological characters were assessed and measured from herbarium material. Flowers and immature fruit were rehydrated in warm Pohl’s solution (Pohl 1965) prior to dissection and measurement. Other characters were measured directly from the herbarium specimens. For the new species from Ecuador, flower colour, habit and habitat information were taken from label data and field observations by Gwilym Lewis and Bente Klitgaard (pers. comm.); for the other three species, these features were described from label data. Reproductive status is indicated with the following abbreviations (alone or in combination): fl, flowering; fr, fruiting; im, immature; st, sterile. Illustrations were made at the National Museum of Natural History, Washington DC, USA by Mattias S. Lanas (*Cynometrasteyermarkii*), Katherine Rudebusch (*C.tumbesiana*) and Alice Tangerini (*C.cerebriformis* and *C.dwyerii*) from herbarium specimens. Two of the new species are described based on a single collection: in both cases, the taxa are morphologically and geographically distinct and, after extensive herbarium study, the author is confident that the available material, while limited, is sufficient to distinguish two new entities at the species level.

## Part I: A new circumscription of the genus

Linnaeus established the genus *Cynometra* and included two species from southeast Asia in the first edition of Species Plantarum, *Cynometracauliflora* L. and *C.ramiflora* L. (Linnaeus, 1753). This original concept of *Cynometra* was of a genus distinguished by compound leaves, four reflexed sepals, five equal petals and by single-seeded, indehiscent pods with thick and tuberculate valves (Linnaeus’ description does not explicitly mention ‘indehiscent pods’, but it is a character present in both of his original species). For nearly the next century, this circumscription was generally followed by taxonomists around the world. The limits of the genus were first expanded by the publication of two new species from the Indo-Pacific region: *Cynometrapolyandra* Roxb., described in 1820 from Indian material and *Cynometragrandiflora* A.Gray, described in 1854 from Fijian specimens. These species differed most notably from the traditional understanding of *Cynometra* by having more numerous stamens (35–40 in *C.grandiflora* and approximately 50 in *C.polyandra*; Roxburgh 1820, Gray 1854). Bentham (1865a) later described the African species *C.floribunda* Benth. and *C.laxiflora* Benth., species with large, petaloid bracteoles and only two showy petals which he placed in a new subgenus, *Hymenostegia* (the fruit of the two species also differs from *Cynometra*, but these were unknown at the time). Scheffer (1876) established the segregate genus *Maniltoa* after viewing collections from New Guinea; believing the material to be conspecific, he included a single species, *Maniltoagrandiflora* (A.Gray) Scheff. (based on *Cynometragrandiflora* A.Gray). While the most apparent difference between the new genus *Maniltoa* and typical *Cynometra* was the number of stamens, Scheffer (1876) argued that the genus was distinct based on the size of the flowers, the number of seeds, caducous filiform stipules, the presence of a tendril terminating the leaf rachis and bracts subtending each pedicel in the inflorescence. He does not include or reference *C.polyandra*, another polyandrous species. *Maniltoa* was not universally accepted at first and Taubert’s (1894) treatment of the Leguminosae for Engler’s Die Natürlichen Pflanzenfamilien treated *Maniltoa* as a section of *Cynometra* (along with the sections *Eucynometra*, *Hymenostegia* and *Pseudocynometra*). A few years later, however, Harms (1897) broke apart this broad concept of *Cynometra*. He elevated *Hymenostegia* to the rank of genus and restored Maniltoaby combining it withsectionPseudocynometra (consisting of a single species, *C.polyandra*) on the basis of greater stamen number and the presence of prominent buds enclosed in imbricate scarious bract scales (Harms 1897, 1902, 1919). This understanding of *Cynometra* and *Maniltoa* in the Indo-Pacific was further refined by Knaap-Van Meeuwen (1970) who sought to better define the limits between the two genera – she again placed greatest significance on the stamen number, placing those with 8–13 stamens in *Cynometra* and those with 15–80 in *Maniltoa*. While many authors have followed this delimitation (Verdcourt 1979; Hou et al 1996), some have continued to question the distinctionof the two genera (Soerianegara 1993; Pan et al. 2010).

In Africa, misunderstanding of the delimitation of *Cynometra* persisted, even after Harms’ (1897) treatment and the turn of the century marked the beginning of a period of rapid taxonomic expansion in both the number of species and, consequently, the characters describing the generic limits of *Cynometra*. As explained (in great detail) by Léonard (1951), the first few species described in Africa generally matched the Linnaean concept (e.g. *Cynometravogelii* Hook.f. in 1849 from Nigeria; *C.mannii* Oliv. in 1871 from Cameroon). However, with the description of *Cynometrasessiliflora* Harms, Harms (1899) expanded the understanding of *Cynometra* to include species with five erect sepals (instead of four reflexed sepals) and, most importantly for the continued confusion regarding the genus, dehiscent pods with thin, flat valves. Not long after, the publication of *Cynometraalexandri* C.H.Wright, *C.gilletii* De Wild., *C.laurentii* De Wild., *C.lujae* De Wild., *C.oddonii* De Wild. and *C.pedicellata* De Wild. added more species with five sepals and dehiscent fruits and further expanded the genus to include species with an intrastaminal disc and numerous ovules (Wright 1902; De Wildeman 1904, 1905, 1906, 1907). Harms (1907) also continued to expand the generic conception of *Cynometra* in Africa to include species with alternate leaflets, distichous flowers and a stipe fused with the receptacle wall when he described *C.leptantha* Harms, *C.multijuga* Harms and *C.pierreana* Harms. *Cynometrabrachyura* Harms, expanded the genus to again include species with only two petals (Harms 1913). As a result, species with all manner of combinations of these characters were placed in *Cynometra* (see Baker 1930 and Pellegrin 1949). While many taxonomists, working in Africa, noted the heterogeneity of the genus, it was not until Léonard undertook a thorough and careful revision of the African species that a coherent circumscription of *Cynometra* began to emerge (Baker 1930; Lebrun 1933; Aubréville 1936; Léonard 1951, 1957).

Léonard’s work (1951, 1957) was a major advancement in revising the boundaries of *Cynometra* and its allies in Africa, which had become so broad as to make genera nearly indistinguishable. Léonard (1951) removed from *Cynometra* any taxa with the stipe of the ovary adnate to the hypanthium or eccentrically inserted (species with a free, central stipe but alternate leaflets with translucent dots were moved to *Gilletiodendron* Vermoesen). To accommodate his revised concept of the genus, Léonard (1951, 1957) described a new genus, *Lebruniodendron* J.Léonard, resurrected the genera *Gilletiodendron* Vermoesen and *Zenkerella* Taub. and transferred numerous species out of *Cynometra* and into the genera *Hymenostegia* (Benth.) Harms, *Plagiosiphon* Harms, *Schotia* Jacq. and *Scorodophloeus* Harms. He placed the remaining *Cynometra* into three informal groups; they share a centrally inserted stipe, opposite leaflets, five equal petals and imbricate sepals, but differ notably in characters such as inflorescence structure, presence or absence of a staminal disc, presence or absence of foliar glands and fruit shape and dehiscence. Léonard (1951) acknowledged that the genus remained heterogenous – the species of ‘group one’ resembled ‘typical *Cynometra*’, but the others were possibly misplaced. Later, he further assessed the genera of African Amherstieae and Cynometreae, revising the tribal and generic limits using characters from germination mode, seedling architecture and wood structure, amongst others. (Léonard 1957). A thorough examination of the genus *Cynometra*, unfortunately, was not included in that otherwise exhaustive study. Léonard and others recognised that, as contemporarily circumscribed, the genus was likely polyphyletic and would require further revisionary studies at a global scale (Aubréville 1968, 1970; Breteler 1996; Léonard 1996).

An early morphological cladistic analysis by Temu (1990) provided the first phylogenetic evidence for the non-monophyly of African *Cynometra*. The results showed two distinct clades of *Cynometra* in tropical Africa, one of which included *Maniltoa*. Later morphological and molecular phylogenetic analyses, albeit with limited sampling of *Cynometra* and *Maniltoa*, also found no evidence to support monophyly of *Cynometra* and suggested that it may be paraphyletic with respect to *Maniltoa* (Bruneau et al. 2001, 2008). More recent studies which included a greater taxon sampling from *Cynometra* and *Maniltoa* have found support for two clades of *Cynometra*, one consisting of exclusively East African species and the other containing species from the rest of the genus plus *Maniltoa* (Mackinder et al. 2013; Radosavljevic et al. 2017; de la Estrella et al. 2018). Radosavljevic et al. (2017), which featured 47 accessions from 36 species of *Cynometra* and *Maniltoa*, recovered two strongly supported clades of *Cynometra* sensu lato, which are each diagnosed by several morphological characters (Figure 1). One clade (*Cynometra* clade A of Radosavljevic et al. 2017) is comprised of exclusively east African species and is grouped as sister to a clade composed of *Dicymbe* Spruce ex Benth. and *Polystemonanthus* Harms with strong support. The other clade, which includes the type species *Cynometracauliflora* L., contains *Cynometra* species from the Neotropics, the remaining Afrotropical species and the Indo-Pacific species (*Cynometra* clade B of Radosavljevic et al. 2017). *Maniltoa* is nested within clade B and is also recovered as non-monophyletic. This clade of *Cynometra* and *Maniltoa* is placed in a clade with *Zenkerella* Taub., *Normandiodendron* J. Léonard and the *Scorodophloeus* clade, again with strong support. The species of *Cynometra* clade A are characterised by the absence of foliar extra-floral nectaries, paniculate inflorescences (with two exceptions), articulated pedicels, a well-developed intrastaminal disc, 2–4 ovules and dehiscent fruit. *Cynometra* clade B is characterised by the presence of foliar extra-floral nectaries (in most species), racemose inflorescences, simple pedicels, the absence of an intrastaminal disc, 1–2 ovules and indehiscent fruit. The species of *Cynometra* clade A correspond to Léonard’s (1951) groups 2 and 3 while *Cynometra* clade B, which contains the generitype *Cynometracauliflora* L., corresponds to group 1. Léonard (1951, 1996) predicted as much; he referred to group 1 as ‘typical *Cynometra*’ and believed that *Cynometraalexandri* and *C.hankei* Harms were likely misplaced. De la Estrella et al. (2018), who sampled broadly across Detarioideae, placed *Cynometra* and *Maniltoa* in a re-circumscribed Amherstieae.

To reconcile the discrepancy between the current classification of *Cynometra* and *Maniltoa* and our understanding of their evolutionary relationships, I present here an emended generic circumscription of *Cynometra* that excludes the species of *Cynometra* clade A and includes the species formerly placed in *Maniltoa*. A treatment that proposes a new genus for the species of *Cynometra* clade A is in preparation.

### Taxonomic treatment

#### *Cynometra* L. Sp. Pl. 1:382. 1753.

Syn. *Iripa* Adans., Fam. 2: 508. 1763. *Cynomora* R.Hedw. Gen. Pl. [R. Hedwig]. 1806. Type. *Cynometracauliflora* L. (lectotype, designated by A.S. Hitchcock, in Hicthcock and Green 1959, pg. 152)

*Metrocynia* Thouars, Gen. Nov. Madagasc. 22. 1806. Type. *Metrocyniacommersoniana* DC.

*Maniltoa* Scheff., Ann. Jard. Bot. Buitenzorg 1: 20. 1876. Type. *Cynometragrandiflora* A.Gray.

*Schizosiphon* K.Schum., Fl. Kais. Wilh. Land 101. 1889. *Schizoscyphus* K.Schum ex Taubert, *nom. superfl.* Bot. Centralbl. 41: 265. 1890. Type. *Schizosiphonrosea* K. Schum.

*Pseudocynometra* Kuntze in Post & Kuntze, Lex. Gen. Phan. Phan. 464. 1903. Type. *Cynometrapolyandra* Roxb.

**Trees** or **shrubs** (infrequent), evergreen (rarely deciduous), buttressed or not, growth flush-wise, new growth flaccid and whitish or reddish at emergence, becoming green with maturity, vegetative buds covered in a series of imbricate scales. *Stipules* lateral, free, linear or filamentous, early caducous, scars typically not visible on mature growth. **Leaves** pulvinate, petiolate, rachis (if present) terete or caniculate, often terminating in a filiform outgrowth, axes glabrous or pubescent, paripinnate with 1–16 pairs of opposite leaflets (rarely unifoliolate); leaflets petiolulate but sometimes appearing sessile because of decurrent lamina, blade lanceolate, ovate, elliptic, oblong, obovate, oblanceolate or trapeziform, symmetrical to strongly asymmetrical, glabrous or sparsely pubescent abaxially, glabrous adaxially (rarely with sparse pubescence along midvein), margins entire, apex broadly obtuse to acuminate, retuse or emarginate, mucronate, base oblique, distal margin decurrent to petiolule; laminar nectaries usually present, abaxial, submarginal, embedded in laminar surface, shallow, without noticeably raised edges. **Inflorescences** axillary or ramiflorous (rarely cauliflorous), racemose, 1–2(–3), per axil, buds enclosed by imbricate bracts, appearing conical, ovoid or cigar-shaped in silhouette; pedicels simple (not articulated), filamentous in anthesis, accrescent and lignified in fruit; bracts enclosing inflorescence during development prior to emergence, imbricate, distichous, deciduous or persistent; bracteoles not enclosing buds, paired, inserted along proximal half of pedicel, opposite or subopposite, caducous. **Flowers** bisexual, actinomorphic; hypanthium present (sometimes indistinct), either turbinate-campanulate and short or tubular and partially to completely enveloping ovary; disc absent; sepals (3–)4(–5), reflexed at anthesis, unequal, deltoid, ovate, elliptic or oblong; petals 5, crumpled or smooth, incurved, erect or horizontal, equal, linear, oblong, or oblanceolate, with rounded or acute apices; stamens (8–)10–80, filaments free or briefly connate basally; anthers dorsifixed, versatile, longitudinally dehiscent; ovary inserted +/- centrally, occasionally eccentric, free, short-stipitate or subsessile, pubescence varied but rarely glabrous, light green or pinkish, often turning red post anthesis, ovules 1 (2); style eccentric, glabrous; stigma capitate. **Legumes**indehiscent, splitting along suture after germination due to action of emerging shoot, orbicular to oblong, often laterally compressed, smooth to deeply fissured, often apiculate (in some taxa only when immature), valves 0.5–4.0 mm thick, cork-like in several species, remaining attached to cotyledons during and after germination. **Seeds** 1–2, enclosed in fruit until germination. **Seedling** germination epigeal.

The emended description above reflects the merger of *Maniltoa* and *Cynometra* s.s. and the exclusion of the east African taxa with dehiscent pods, paniculate inflorescences and articulated pedicels (Tables 1, 2). While many taxa in the former *Maniltoa* are no doubt distinctive and, in many cases, striking when compared to the more unassuming *Cynometra*, a close examination of their morphological characteristics reveals a strong similarity. The number of stamens has been perhaps the most distinctive and most often cited character separating the two genera. *Cynometra* has long been known to have only 10 stamens, while *Maniltoa* has been traditionally treated as having anywhere from 15–80 stamens. Indeed, this proliferation in stamen number is rare in the Detarioideae. However, a closer look at the Indo-Pacific *Cynometra* reveals several examples of species which regularly have as few as eight stamens (*Cynometraglomerulata* Gagnep.) or as many as 12 (*Cynometrakatikii* Verdc.) and some with variation across the range within a species (8–10 stamens in *Cynometracauliflora* L. and 10–15 stamens in *Cynometraramiflora* L.; Knaap-van Meeuwen 1970, Verdcourt 1979). So, while the majority of species, placed within *Cynometra*, do have 10 stamens, there is lability amongst this trait in the taxa of the region.

**Table 1.** Species excluded from *Cynometra*. Distributions correspond to regions given in text. Species may occupy only a part of the overall region. MTA = mainland tropical Africa.

Species	Distribution
*Cynometraalexandri* C.H. Wright	MTA
*Cynometraananta* Hutch. & Dalziel	MTA
*Cynometrabrachyrrhachis* Harms	MTA
*Cynometraengleri* Harms	MTA
*Cynometrafilifera* Harms	MTA
*Cynometrafischeri* Baker f.	MTA
*Cynometragilletii* De Wild.	MTA
*Cynometragillmanii* J. Léonard	MTA
*Cynometragreenwayi* Brenan	MTA
*Cynometrahankei* Harms	MTA
*Cynometraleonensis* Hutch. & Dalziel	MTA
*Cynometralongipedicellata* Harms	MTA
*Cynometralujae* De Wild.	MTA
*Cynometranyangensis* Pellegr.	MTA
*Cynometraoddonii* De Wild.	MTA
*Cynometrapalustris* J. Léonard	MTA
*Cynometrapedicellata* De Wild.	MTA
*Cynometrasessiliflora* Harms	MTA
*Cynometrasuaheliensis* (Taub.) Baker f.	MTA
*Cynometraulugurensis* Harms	MTA
*Cynometrawebberi* Baker f.	MTA

**Table 2.** Accepted species of *Cynometra*. Taxonomy follows: Knaap Van-Meeuwen 1970; Léonard 1951; Dwyer 1958. New combinations and new species presented in text are not included. Distributions correspond to regions given above. Species may occupy only a part of the overall region. COM = Comoros Islands; INP = Indo-Pacfic; MAD = Madagascar; MTA = mainland tropical Africa; NEO = Neotropics.

Species	Distribution	Species	Distribution
*Cynometraabrahamii* Du Puy & R.Rabev.	MAD	*Cynometralongifolia* Huber	NEO
*Cynometraamericana* Vogel	NEO	*Cynometralukei* Beentje	MTA
*Cynometraankaranensis* Dupuy & R.Rabev.	MAD	*Cynometralyallii* Baker	MAD
*Cynometraaurita* R.Vig.	MAD	*Cynometramacrocarpa* A.S.Tav.	NEO
*Cynometrabauhiniifolia* Benth.	NEO	*Cynometramadagascariensis* Baill.	MAD
*Cynometrabeddomei* Prain	INP	*Cynometramalaccensis* Meeuwen	INP
*Cynometrabourdillonii* Gamble	INP	*Cynometramannii* Oliv.	MTA
*Cynometrabrachymischa* Harms	INP	*Cynometramarginata* Benth.	NEO
*Cynometracapuronii* Du Puy & R.Rabev.	MAD	*Cynometramarleneae* A.S.Tav.	NEO
*Cynometracauliflora* L.	INP	*Cynometramayottensis* Labat & O.Pascal	COM
*Cynometracloiselii* Drake	MAD	*Cynometramegalophylla* Harms	MTA
*Cynometracommersoniana* Baill.	MAD	*Cynometramicroflora* R.S.Cowan	NEO
*Cynometracongensis* De Wild.	MTA	*Cynometraminutiflora* F.Muell.	INP
*Cynometracopelandii* (Elmer) Elmer	INP	*Cynometramirabilis* Meeuwen	INP
*Cynometracraibii* Gagnep.	INP	*Cynometranovoguineensis* Merr. & L.M.Perry	INP
*Cynometracrassifolia* Benth.	NEO	*Cynometraoaxacana* Brandegee	NEO
*Cynometracubensis* A.Rich.	NEO	*Cynometraparvifolia* Tul.	NEO
*Cynometracuneata* Tul.	NEO	*Cynometrapervilleana* Baill.	MAD
*Cynometradauphinensis* Dupuy & R.Rabev.	MAD	*Cynometrapolyandra* Roxb.	INP
*Cynometradongnaiensis* Pierre	INP	*Cynometraportoricensis* Krug & Urb.	NEO
*Cynometraduckei* Dwyer	NEO	*Cynometraramiflora* L.	INP
*Cynometraelmeri* Merr.	INP	*Cynometraretusa* Britton & Rose	NEO
*Cynometrafalcata* A.Gray	INP	*Cynometrasakalava* Du Puy & R.Rabev.	MAD
*Cynometrafissicuspis* (Pittier) Pittier	NEO	*Cynometrasanagaensis* Aubrev.	MTA
*Cynometrafloretii* Labat & O.Pascal	COM	*Cynometraschlechteri* Harms	MTA
*Cynometraglomerulata* Gagnep.	INP	*Cynometraschottiana* Hochr.	NEO
*Cynometragrandiflora* A.Gray	INP	*Cynometrasimplicifolia* Harms	INP
*Cynometrahemitomophylla* (Donn.Sm.) Rose	NEO	*Cynometraspruceana* Benth.	NEO
*Cynometrahondurensis* Dwyer	NEO	*Cynometrastenopetala* Dwyer	NEO
*Cynometrahostmanniana* Tul.	NEO	*Cynometratravancorica* Bedd.	INP
*Cynometrahumboldtiana* Stergios	NEO	*Cynometratrinitensis* Oliv.	NEO
*Cynometrainsularis* A.C.Sm.	INP	*Cynometravogelii* Hook.f.	MTA
*Cynometrairipa* Kostel.	INP	*Cynometrawarburgii* Harms	INP
*Cynometrakatikii* Verdc.	INP	*Cynometrawhitfordii* Elmer	INP
*Cynometraletestui* (Pellegr.) J.Léonard	MTA	*Cynometrayokotai* Kaneh.	INP
*Cynometralongicuspis* Ducke	NEO	*Cynometrazeylanica* Kosterm.	INP

Several other characteristics of *Maniltoa* that supposedly distinguish it from *Cynometra* are also incorrect. The first is the presence of conspicuous ‘bract covered’ vegetative and reproductive buds (Scheffer 1876, Harms 1902, 1919). In some species of *Maniltoa*, these buds can be many centimetres long and several centimetres in diameter. The scales themselves are often tan or brown, but can be whitish or pinkish and are often covered with parallel striations running longitudinally along the surface. These types of buds are also present in *Cynometra*, although they are much smaller, in some cases only 4 or 5 mm long. Likewise, the scales are similar in shape, vestiture and surface texture. Another general characteristic used to separate the two genera has been the sturdiness of the inflorescence rachis in *Maniltoa* (Knaap-van Meeuwen 1970). However, this is likely related to the overall general difference in size of the flowers and inflorescence between the two genera: *Maniltoa* tend to have larger buds, bracts, inflorescences and individual flowers. The larger inflorescences of the *Maniltoa* species may be an adaptation to mammal pollination as there are reports of marsupials and bats visiting the flowers of *Maniltoa* species in Australia and the Pacific Islands (Marshall 1985, Endress 1994). Scheffer (1876) cited the presence of a tendril terminating the leaf rachis as a distinguishing character of *Maniltoa*, but Dwyer (1958) notes that this trait is present in several *Cynometra*. Finally, differences in wood anatomy given by Knaap-van Meeuwen (1970) have been found to be insufficiently distinct from one another and overlapping in range (Soerianegara 1993; Pan et al. 2010).

The most clearly distinguishing feature of the newly emended *Cynometra* with respect to the taxa here excluded (*Cynometra* clade A) is the indehiscent pod. Indeed, if one examines the taxa misplaced in *Cynometra* over the years, they nearly all share the characteristic of dehiscent pods. Unfortunately, Linnaeus makes no mention of the nature of the pods’ dehiscence. Likewise, subsequent treatments were vague. Bentham (1865b), Taubert (1894) and Harms (1897) all described the pods as ‘two-valved’ without further elaboration. Léonard (1951) and Aubréville (1968, 1970) cite the pods of ‘typical *Cynometra*’ as dehiscent or indehiscent, likely owing to the fruit of *Cynometramannii*, the valves of which are not dehiscent but, upon pressing and drying, often split and rupture in several places along axes roughly perpendicular to the sutures. A similar occurrence is observed in the Neotropical species *Cynometrabauhiniifolia* Benth. When Harms (1899) described *C.sessiliflora*, he set a precedent regarding the pod characters that contributed to the imprecise delimitation of *Cynometra* and subsequently led to the taxonomic confusion surrounding *Cynometra* in Africa.

With this new circumscription, the two genera are united and share radially symmetric flowers, bract covered buds (with bracts persisting on the inflorescence), flowers with early caducous bracteoles not enveloping the flowers and indehiscent fruits (which may play a role in dispersal as many taxa are associated with alluvial habitats or appear as drift ‘fruit’ in coastal environments; Ridley 1930, Léonard 1951, Clarke et al. 2001, Tomlinson 2016). The new classification changes the overall distribution of the taxa. Prior to this, *Cynometra* was a genus with its diversity distributed somewhat evenly across the American, African and Asian tropics. However, with the exclusion of many of the African species and the inclusion of the former *Maniltoa*, *Cynometra* is now a genus whose primary centre of diversity is the Indo-Pacific region, with a secondary centre in the Neotropics and the majority of Afrotropic taxa restricted to Madagascar.

## Part II: Reduction of *Maniltoa* into *Cynometra*

The generic rearrangements require new combinations to reflect the merging of *Maniltoa* into *Cynometra*. For *Maniltoa*, in most cases, the taxonomy proposed by Knaap-van Meeuwen (1970) has been followed, but for some species limits, the interpretations of Verdcourt (1979) have been followed; such deviations are noted. Species, for which a combination already exists in *Cynometra*, are not listed (Knaap-van Meeuwen 1970). In total, 18 new combinations and one new name are proposed. A lectotype is designated for *Maniltoamegalocephala* Harms and a neotype is designated for *Maniltoapeekelii* Harms.

### 1. Cynometra basifoliola

Taxon classificationAnimaliaFabalesFabaceae

(Verdc.) Rados. comb. nov.

urn:lsid:ipni.org:names:77199243-1

#### Basionym.

*Maniltoabasifoliola* Verdc., Kew Bull. 37: 129. 1982. Type. PAPUA NEW GUINEA. Madang province: Madang subprovince [district], cleared area utilized as a gravel pit on north side of Madang Usino Highway on banks of Gogol River, 145 37 E, 05 15 S, 100 m alt., 25 April 1979, *S.H. Somer & P. Katik LAE 75185* (holotype: K; isotypes: BM, L, M).

#### Notes.

The arrangement of leaflets for which this species is named (i.e. the basal-most pair of leaflets inserted just above petiole and separated from terminal pair of leaflets by a relatively long rachis) is unusual amongst the species that were formerly included in *Maniltoa*; however some species of *Cynometra* have a similar arrangement (e.g. *Cynometrasakalava* Du Puy & R.Rabev from Madagascar).

### 2. Cynometra brassii

Taxon classificationAnimaliaFabalesFabaceae

(Merr. & L.M.Perry) Rados. comb. nov.

urn:lsid:ipni.org:names:77199244-1

#### Basionym.

*Maniltoabrassii* Merr. & L.M.Perry, J. Arnold Arbor. 23: 398. 1942. Type. BRITISH NEW GUINEA [PAPUA NEW GUINEA]. Central Division: U-uma River, 14 May 1926, [fl., imm. fr.], *L. J. Brass 1428*, (holotype: A; isotype: K).

#### Notes.

The protologue lists the collection year as 1928, however 1926 appears to be the correct date. While the label affixed to the holotype does list the collection date as “14 May 1928”, this appears to be a transcription error. The handwritten slip attached to the holotype, presumably filled out by the collector Brass, gives the date as “14/5/26”. Additionally, the printed label is titled “Arnold Arboretum Expedition, 1925–1926”. The label on the isotype also has the collection date as 14 May 1926.

### 3. Cynometra browneoides

Taxon classificationAnimaliaFabalesFabaceae

(Harms) Rados. comb. nov.

urn:lsid:ipni.org:names:77199245-1

#### Basionym.

*Maniltoabrowneoides* Harms, Notizbl. Königl. Bot. Gart. Berlin 3: 190. 1902. Synonym. *Pseudocynometrabrowneoides* (Harms) Kuntze, Deutsche Bot. Monatsschr. 21: 173. 1903. Type. [INDONESIA]. Java. [West Java: Bogor Botanic Gardens], 1880–1882, [fl], *H. O. Forbes 1204a* (holotype: B, destroyed; isotype: BM).

#### Synonym.

*Maniltoagemmipara* Scheff. ex Backer, Voorl. Schoolfl. Java 104: in clavi. 1908. Type. [INDONESIA]. Cultivated at Weltevreden (Djakarta), 1908, *Backer s.n.* (holotype: L, fide Knaap-van Meeuwen 1970).

#### Notes.

According to Knaap-van Meeeuwen (1970, p. 42), the type material for *Maniltoabrowneoides* came from a cultivated specimen grown at Bogor Botanical Gardens from seeds or seedlings collected by Forbes on New Guinea, however no citation is given as the source for this information. The holotype for *M.gemmipara* is listed as being at Leiden by Knaap-van Meeuwen, however there are no specimens there annotated as such. There are two specimens, however, from Java collected in 1908 that seem to match the details in the protologue. These are filed as *M.gemmipara* and have the registration numbers L.3886447 and L.3886448.

### 4. Cynometra cynometroides

Taxon classificationAnimaliaFabalesFabaceae

(Merr. & L.M.Perry) Rados. comb. nov.

urn:lsid:ipni.org:names:77199246-1

#### Basionym.

*Maniltoacynometroides* Merr. & L.M.Perry, J. Arnold Arbor. 23: 398. 1942. Type. BRITISH NEW GUINEA [PAPUA NEW GUINEA]. Palmer River, 2 mi. below junction Black River, 100m alt., Jun 1936, [fr], *L. J. Brass 6903* (holotype: A; isotypes: BM, BO, BRI, L).

### 5. Cynometra fortuna-tironis

Taxon classificationAnimaliaFabalesFabaceae

(Verdc.) Rados. comb. nov.

urn:lsid:ipni.org:names:77199247-1

#### Basionym.

*Maniltoafortuna-tironis* Verdc., Kew Bull. 32 (1): 243. 1977. Type. PAPUA NEW GUINEA. Central District [Central Province]: Rouna Falls area, forest by tributary of R. Laloki, 300 m alt., 2 Jan. 1976, *B. Verdcourt*, *C.R. Huxley*, *& Dodd 4899* (holotype: K; isotypes: LAE, UPNG).

### 6. Cynometra lenticellata

Taxon classificationAnimaliaFabalesFabaceae

(C.T.White) Rados. comb. nov.

urn:lsid:ipni.org:names:77199248-1

#### Basionym.

*Maniltoalenticellata* C.T.White, J. Arnold Arbor. 8: 130. 1927. Type. PAPUA [PAPUA NEW GUINEA]. Northern Division: Sageri, July 1922, [fl], *C. E. Lane-Poole 203* (holotype: BRI; isotype: A).

### 7. Cynometra lenticellata var. villosa

Taxon classificationAnimaliaFabalesFabaceae

(Verdc.) Rados. comb. nov.

urn:lsid:ipni.org:names:77199249-1

#### Basionym.

Maniltoalenticellatavar.villosa Verdc., Kew Bull. 32 (1): 241. 1977. Type. TERRITORY OF NEW GUINEA [PAPUA NEW GUINEA]. Morobe district [Morobe province]: Lae subdistrict [Lae district], Kassam Pass, 6°20'S, 146°00'E, 3500 ft alt., [fl], *J.S. Womersley & J. Vandenberg NGF 37192* (holotype: LAE; isotypes: A, BISH, BO, BRI, CANB, K, L, NSW, PNH, SING, UPNG, US)

### 8. Cynometra mariettae

Taxon classificationAnimaliaFabalesFabaceae

(van Meeuwen) Rados. comb. nov.

urn:lsid:ipni.org:names:77199250-1

#### Basionym.

*Maniltoamariettae* van Meeuwen, Blumea 18 (1): 37. 1970. Type. TERRITORY OF NEW GUINEA [PAPUA NEW GUINEA]. Morobe District [Morobe Province]: Yalu, 6°36'S, 146°52'E, 50 ft alt., Jul 1944, [fl], *C.T. White*, *H.E. Dadswell*, *& L.S. Smith*, *NGF 1661* (holotype: BRI; isotype: CANB).

### 9. Cynometra megalocephala

Taxon classificationAnimaliaFabalesFabaceae

(Harms) Rados. comb. nov.

urn:lsid:ipni.org:names:77199251-1

#### Basionym.

*Maniltoamegalocephala* Harms, Bot. Jahrb. Syst. 55 (1): 52. 1917. Type. NORDÖSTLICH NEU-GUINEA [PAPUA NEW GUINEA]. Sepik area, 1912–1913, *C. L. Ledermann 7857*, *7895*, *10616* (syntypes: B, destroyed). PAPUA NEW GUINEA. Sepik area, *Harms*, *Bot. Jahrb. Syst. 55* (*1*): *53*, *tab. 2. 1917*. (lectotype, here designated)

#### Notes.

**Figure 2.**
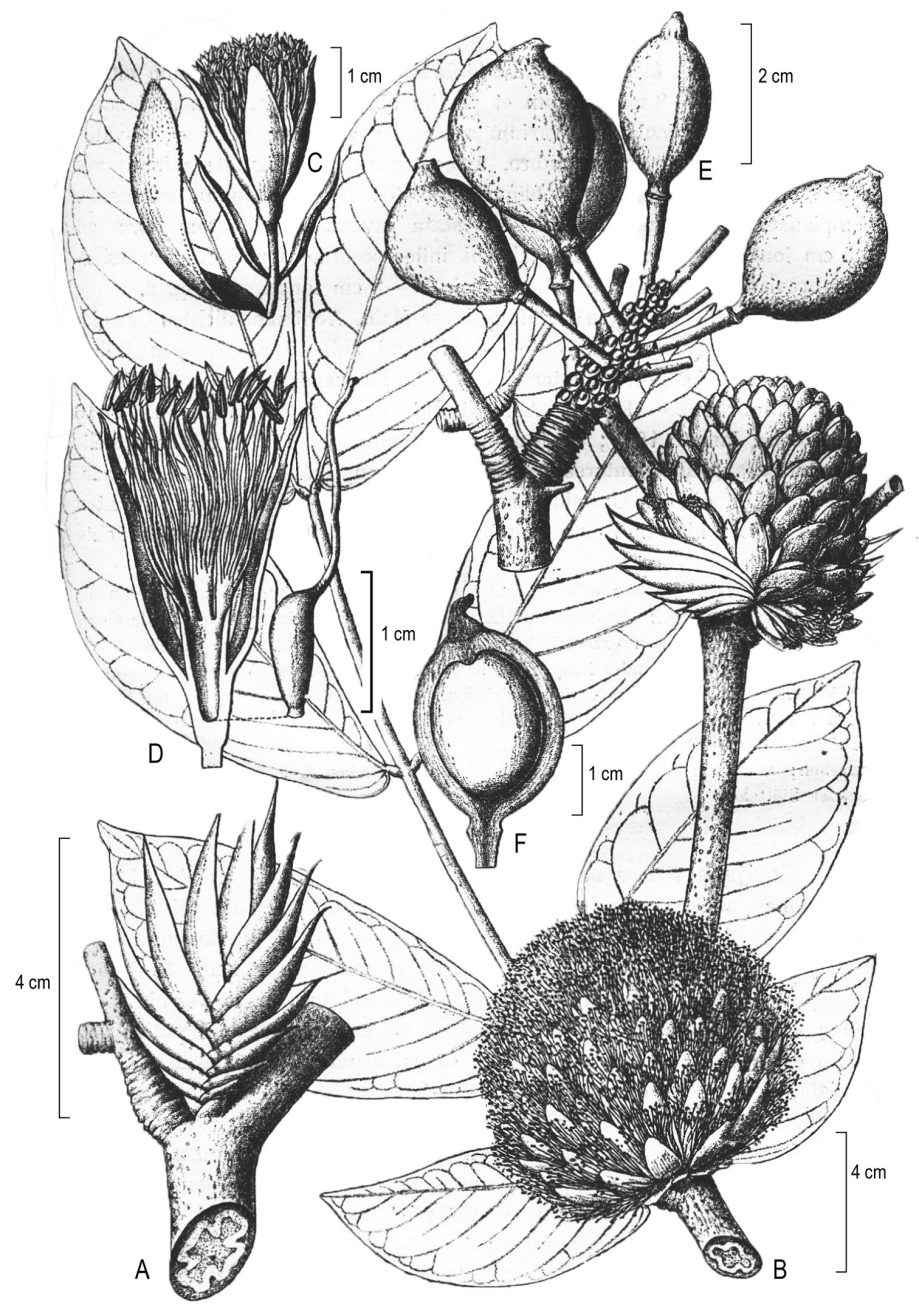
Illustration of *Maniltoamegalocephala* Harms, reproduced from protologue and designated here as the lectotype.

All three syntypes cited in Harms’ original description were destroyed and no isotypes have been located. Therefore plate 2 (tab. 2) from Harms 1917 is designated as the lectotype (Figure 2).

### 10. Cynometra minor

Taxon classificationAnimaliaFabalesFabaceae

(A.C.Sm.) Rados. comb. nov.

urn:lsid:ipni.org:names:77199252-1

#### Basionym.

*Maniltoaminor* A.C.Sm., Sargentia 1: 37. 1942. Type. FIJI. Lau Province: Moala Island, near Maloku, 20–24 Mar 1934, [fl], *A.C. Smith 1333* (holotype: GH; isotype: BISH, K, NY, S, WIS, US).

### 11. Cynometra plurijuga

Taxon classificationAnimaliaFabalesFabaceae

(Merr. & L.M.Perry) Rados. comb. nov.

urn:lsid:ipni.org:names:77199253-1

#### Basionym.

*Maniltoaplurijuga* Merr. & L.M.Perry, J. Arnold Arbor. 23: 398. 1942. Type. NETHERLANDS NEW GUINEA [INDONESIA]. [Papua:] 2 km southwest of Bernhard Camp, Idenburg River, 650 m alt., 3 April 1939, *L.J. Brass & C. Versteegh 13539* (holotype: A; isotype: BO, BRI, L).

### 12. Cynometra psilogyne

Taxon classificationAnimaliaFabalesFabaceae

(Harms) Rados. comb. nov.

urn:lsid:ipni.org:names:77199254-1

#### Basionym.

*Maniltoapsilogyne* Harms, Bot. Jahrb. Syst. 55 (1): 50. 1917. Type. KAISER-WILHELMSLAND [Papua New Guinea]. At Kaulo, ca. 250 m alt., 1 December 1907, *R. Schlechter 16934* (holotype: B; isotypes: A, E, G, K, L, S, Z).

### 13. Cynometra rosea

Taxon classificationAnimaliaFabalesFabaceae

(K.Schum.) Rados. comb. nov.

urn:lsid:ipni.org:names:77199255-1

#### Basionym.

*Schizosiphonroseus* K.Schum., Fl. Kais. Wilh. Land 101. 1889. Syn. *Schizoscyphusroseus* (K.Schum.) Warb., *nom superfl.*, Bot. Jahrb. Syst. 13: 331. 1891. *Maniltoarosea* (K.Schum.) Meeuwen, Blumea 18: 35. 1970. Type. KAISER-WILHELMSLAND [PAPUA NEW GUINEA]. Astrolabebay, Im Hochwalde von Constantinhafen, July [1886–1887], *Hollrung 492* (holotype: B, destroyed; isotypes not found). PAPUA NEW GUINEA. Madang Province: Madang District, Naikum, Josephstaal, 04°45'30"S, 145°00'30"E, 1 Sept. 1958, *K.J. White NGF 10226* (neotype, designated by Knaap-van Meeuwen 1970, p. 35: K; isoneotypes: BRI, CANB).

*Maniltoaurophylla* Harms, Bot. Jahrb. 55: 51. 1917. Type. NORDÖSTLICH NEU-GUINEA [PAPUA NEW GUINEA]. Kameelsrücken, lager G, 6–900 m, Sept. 1912, *C. L. Ledermann 8848* (holotype: B, destroyed; isotypes: WRSL, LE, not located).

#### Notes.

I agree with Verdcourt (1979) that this synonymy is questionable. However, with the original type material destroyed and to avoid creating an unnecessary new combination, I have elected to follow Knaap-van Meeuwen’s treatment here.

### 14. Cynometra schefferi

Taxon classificationAnimaliaFabalesFabaceae

(K.Sch.) Rados. comb. nov.

urn:lsid:ipni.org:names:77199256-1

#### Basionym.

*Maniltoaschefferi* K.Sch., Fl. Kais. Wilh. Land 10. 1889. Syn. *Pseudocynometraschefferi* Kuntze, Deutsche Bot. Monatsschr. 21: 173. 1903. Type. PAPUA NEW GUINEA. Dore, *Teysmann s.n.* (lectotype, designated by Knaap-van Meeuwen 1970, p. 45: L).

*Maniltoahollrungii* Harms, Notizbl. Königl. Bot. Gart. Berlin 3: 189. 1902. Syn. *Pseudocynometrahollrungii* Kuntze, Deutsche Bot. Monatsschr. 21: 173. 1903. Type. KAISER-WILHELMSLAND [PAPUA NEW GUINEA]. Augusta-Station, Aug. 1887, *Hollrung 689* (holotype: B, destroyed; isotypes not found).

### 15. Cynometra schefferi var. peekelii

Taxon classificationAnimaliaFabalesFabaceae

(Harms) Rados. comb. nov.

urn:lsid:ipni.org:names:77199257-1

#### Basionym.

*Maniltoapeekelii* Harms, Bot. Jahrb. Syst. 55 (1): 50. 1917. Syn. Maniltoaschefferivarpeekelii (Harms) Verdc. Type. [PAPUA NEW GUINEA]. Neu-Mecklenburg (New Ireland): Lemakot, Garamate, Strandebene, July 1912, *G. Peekel 841* (holotype: B, destroyed). PAPUA NEW GUINEA. New Ireland District [Province]: [Island of New Ireland], Kavieng Sub-District [District], ca. 26 miles from Kavieng, inland from Lavongai, 2°46'S, 151°2'E, 0 m alt., 24 Jan. 1967, *M.J.E. Coode*, *T. Cropley*, *& P. Katik NGF 29604* (neotype, here designated: K; isoneotypes: A, CANB, L, LAE).

#### Note.

When Verdcourt (1977) published the above name at a new rank, he did not name a neotype. He did cite several representative specimens and specifically mentioned Coode et al NGF 29604 as a specimen he was certain belonged to this species. Given that it is well represented in herbaria and was collected near the original type locality, this specimen has been chosen as the neotype.

### 16. Cynometra steenisii

Taxon classificationAnimaliaFabalesFabaceae

(van Meeuwen) Rados. comb. nov.

urn:lsid:ipni.org:names:77199258-1

#### Basionym.

*Maniltoasteenisii* van Meeuwen, Blumea 18 (1): 40. 1970. Type. TERRITORY OF PAPUA [PAPUA NEW GUINEA]. Northern District [Northern Province]: Tufi subdistrict, near Budi Barracks, 9°32'S, 148°58'E, 75 m alt., 26 August 1954, *R.D. Hoogland 4581* (holotype: CANB).

#### Notes.

Knaap-van Meeuwen incorrectly cites the holotype as being housed in BRI.

### 17. Cynometra steenisii var. rodneyensis

Taxon classificationAnimaliaFabalesFabaceae

(Verdc.) Rados. comb. nov.

urn:lsid:ipni.org:names:77199266-1

#### Basionym.

*Maniltoasteensisii var. rodneyensis* Verdc., Kew Bull. 32 (1): 241. 1977. Type. TERRITORY OF PAPUA [PAPUA NEW GUINEA]. Central District [Central Province]: Abau Subdistrict [Abau District], Cape Rodney, Mori River, 200 ft alt., 20 June 1968, *E. E. Henty NGF 38561* (holotype: LAE; isotypes: A, BISH, BO, BRI, CANB, K, L, NSW, SING).

### 18. Cynometra vestita

Taxon classificationAnimaliaFabalesFabaceae

(A.C.Sm.) Rados. comb. nov.

urn:lsid:ipni.org:names:77199267-1

#### Basionym.

*Maniltoavestita* A.C.Sm., J. Arnold Arbor. 31: 170. 1950. Type. FIJI. Vanua Levu. Mathuata [Macuata] province: east of Lambasa [Labasa], on the southern slopes of Mt Numbuiloa, 3 Nov 1947, [fl], *A.C. Smith 6442* (holotype: A; isotypes: BISH, BRI, K, L, LE, NY, P, S, US).

### 19. Cynometra vitiensis

Taxon classificationAnimaliaFabalesFabaceae

Rados. nom. nov.

urn:lsid:ipni.org:names:77199269-1

#### Basionym.

*Maniltoafloribunda* A.C.Sm., J. Arnold Arbor. 31: 169. 1950, non *Cynometrafloribunda* Benth, Trans Linn. Soc. London 25: 318. 1865. Type. FIJI. Viti Levu. Nandronga-Navosa Province: southern slopes of Nausori Highlands, in drainage of Namosi Creek above Tumbenasolo, 300–400 m alt., 29 May 1947, *A.C. Smith 4588* (holotype: A; isotypes: BISH, BRI, K, L, LE, NY, P, S, US).

#### Notes.

This species is named after the nation of Fiji, where it is endemic, but somewhat common and widespread, occurring on at least six islands (Smith 1985).

## Part III: Four new species of *Cynometra*

**Figure 3.**
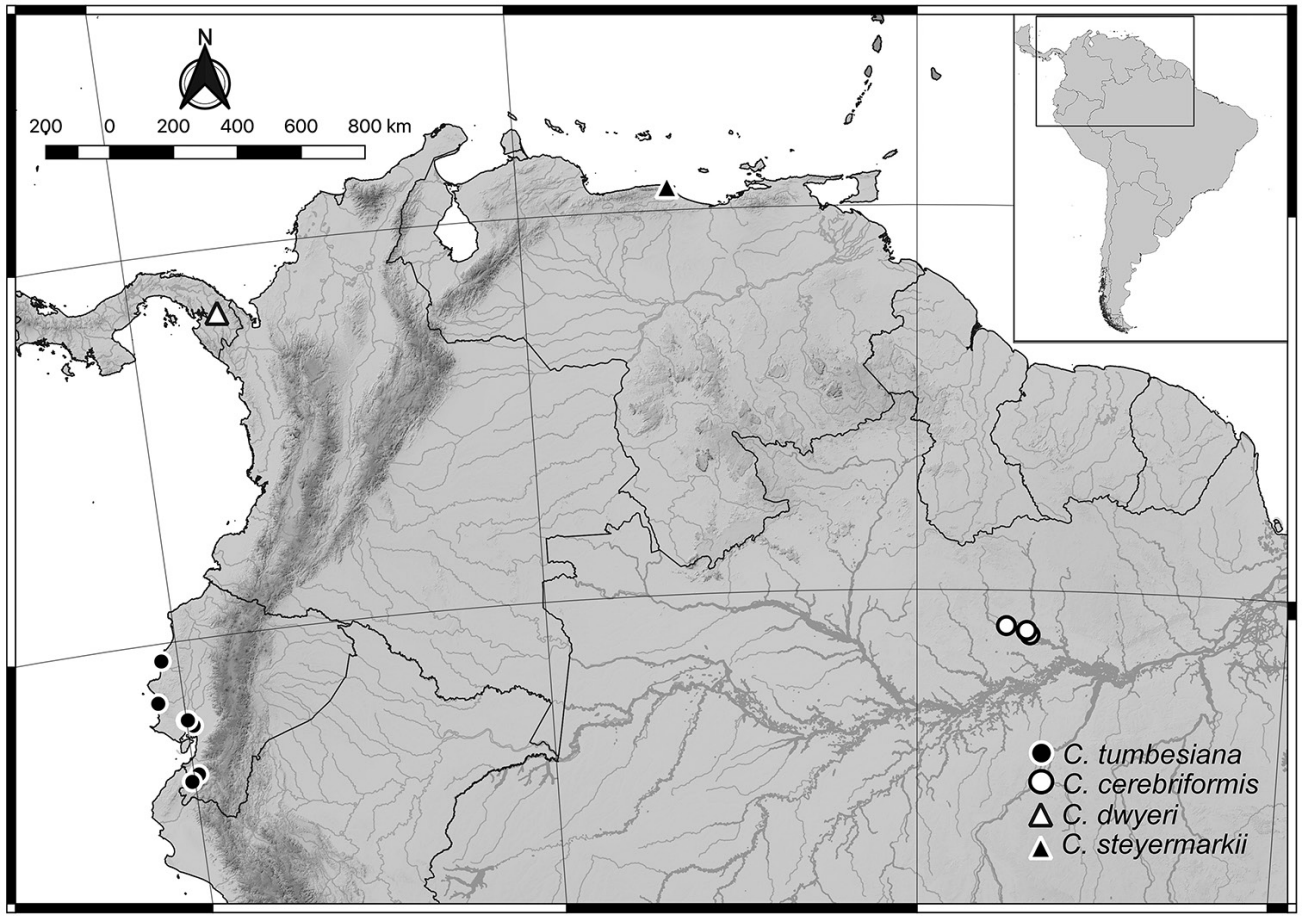
Distribution of *Cynometracerebriformis*, *C.dwyerii*, *C.steyermarkii* and *C.tumbesiana*.

A monograph of the Neotropical species of *Cynometra* was published by Dwyer (1958), but it was limited by the lack of fertile material. In the intervening decades, however, many new collections have been made, particularly in the Amazon basin, the centre of diversity of the genus in the New World. This has made revisionary work more feasible and uncovered previously undocumented diversity. In the context of the ongoing taxonomic and phylogenetic studies, several new species have been discovered in existing herbarium collections. Four new species are described below: *Cynometracerebriformis* sp. nov. from the lower Rio Trombetas in Para State, Brazil; *Cynometradwyerii* sp. nov. from the Darien gap region of Panama; *C.tumbesiana* sp. nov. from the dry tropical forests of Ecuador and Peru; and *C.steyermarkii* sp. nov. from the foothills of the western Cordillera de la Costa in Venezuela (Figure 3).

### 1. Cynometra cerebriformis

Taxon classificationAnimaliaFabalesFabaceae

Rados. sp. nov.

urn:lsid:ipni.org:names:77199270-1

Figures 4 , 5

#### Type.

BRAZIL. Pará: [Mun. Oriximiná] Rio Trombetas, near Cachoeira Porteira, 24 May 1974, [fl.], *D. G. Campbell et al.*, *P22338* (holotype US; isotype F, INPA, MO, NY).

#### Description.

***Tree*** to 20 m tall; bark not seen; branchlets rough, lenticels prominent, bud scale scars partially encircling stems near base, new growth with short scattered pubescence, glabrous or nearly so at maturity. ***Stipules*** not seen. ***Leaves*** bifoliolate, pulvinate, axes glabrous or with sparse pubescence concentrated on adaxial surfaces; petioles 3.0–4.0 mm long, 1.0 mm wide, transversely corrugated; petiolules 0.5–1.0 mm long, 0.5 mm wide, transversely corrugated; leaflets appearing sessile, coriaceous, obelliptic to obovate, asymmetric, primary vein eccentric, proximal side 2.0–2.7 times wider than distal, 3.1–4.5 cm long, 1.1–1.9 cm wide, abaxial surface with scattered raised areas both surfaces glabrous, primary venation pinnate, secondary venation brochidodromous-eucamptodromous, 3–4 basal acrodromous veins, decurrent to primary vein, prominent abaxially, their course barely visible adaxially, tertiary venation difficult to discern on either surface even under magnification, margins entire, apex acute, usually short acuminate (acumen to 4.0 mm), retuse, mucronate, base oblique, acute, distal side narrowly cuneate with margin nearly parallel to midvein for 8.0–10.0 mm, proximal side convex, decurrent to petiolule, laminar glands absent. ***Inflorescence*** an axillary raceme, (1–)2 per axil, bracteate, axes ferrugino-pilose; peduncle 1.0–2.0 mm, rachis 2.0–5.0 mm long, flowers spirally arranged, 2–10 per raceme; pedicels 7.0–10.0 mm and filamentous in anthesis, to 18.0 mm and accrescent in fruit; bracts subtending individual flowers, scale-like, quickly deciduous, brown, broadly elliptical to deltoid, strongly convex 1.5–2.5 mm long, 1.0–1.5 mm wide, striate, abaxial surface pubescent, pubescence denser at base and along margins, glabrous adaxially; bracteoles not seen. ***Flowers*** bisexual, radially symmetric, pentamerous, delicate; hypanthium cupular, 0.7–1.0 mm deep, surrounding basal portion of ovary, fleshy, abaxial surface pubescent, adaxial surface glabrous; sepals 4, imbricate, reflexed, abaxial and adaxial sepals larger than lateral, greenish-white, petaloid, oblong to elliptic, apices acute to rounded, 3.0–4.0 mm long, 1.0–2.0 mm wide, pubescence on abaxial surface near apex, with faint parallel venation; petals 5, equal, white, crumpled texture, curving inwards, oblanceolate, 4.0–5.0 mm long, 1.0–1.5 mm wide, adaxial surface with minute appressed hairs, pinnate venation; stamens 10, filaments free, subequal, 7.0–8.5 mm long, anthers dorsifixed, versatile, longitudinal dehiscence, ellipsoid, to 1.0 mm long, glabrous; ovary centrally inserted, free, stipitate, obliquely elliptical, 2.5–3.0 mm long, 1.5–2.0 mm wide, tomentose, stipe 0.5 mm, style apical, 2.0–2.5 mm long, glabrous, eccentric, curving downwards, stigma capitate. ***Legume*** indehiscent, oblate, rugose, apiculate when immature, 14.2–17.5 mm long, 7.1–9.2 mm wide, 12.1–13.9 mm thick, valves pubescent, wall of pericarp up to 2.5 mm thick, brown. ***Seeds*** 1 per pod, filling locule, dark brown.

**Figure 4.**
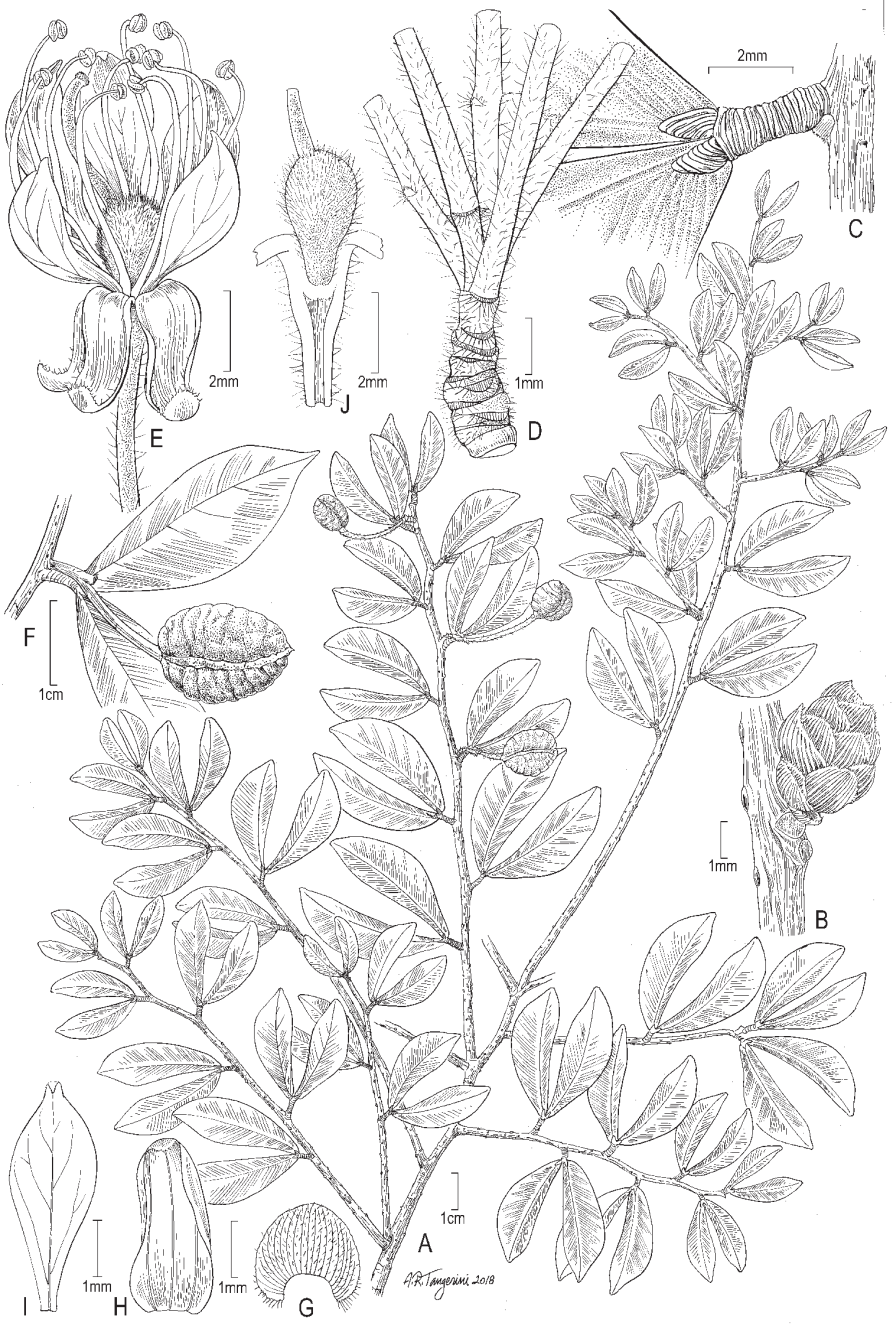
Illustration of *Cynometracerebriformis*. **A** Habit **B** bud with imbricate bracts **C** leaf base showing corrugated petiole and petiolule **D** inflorescence rachis showing bract scars on main axis and remnants of bracteoles on pedicels **E** flower **F** infructescence with single pod **G** bract **H** sepal **I** petal **J** longitudinal section of hypanthium and receptacle; sepals, petals and stamens removed. **A–E, G–J***Campbell et al. P22338*, US **F***G. Martinelli 7016*, US.

**Figure 5.**
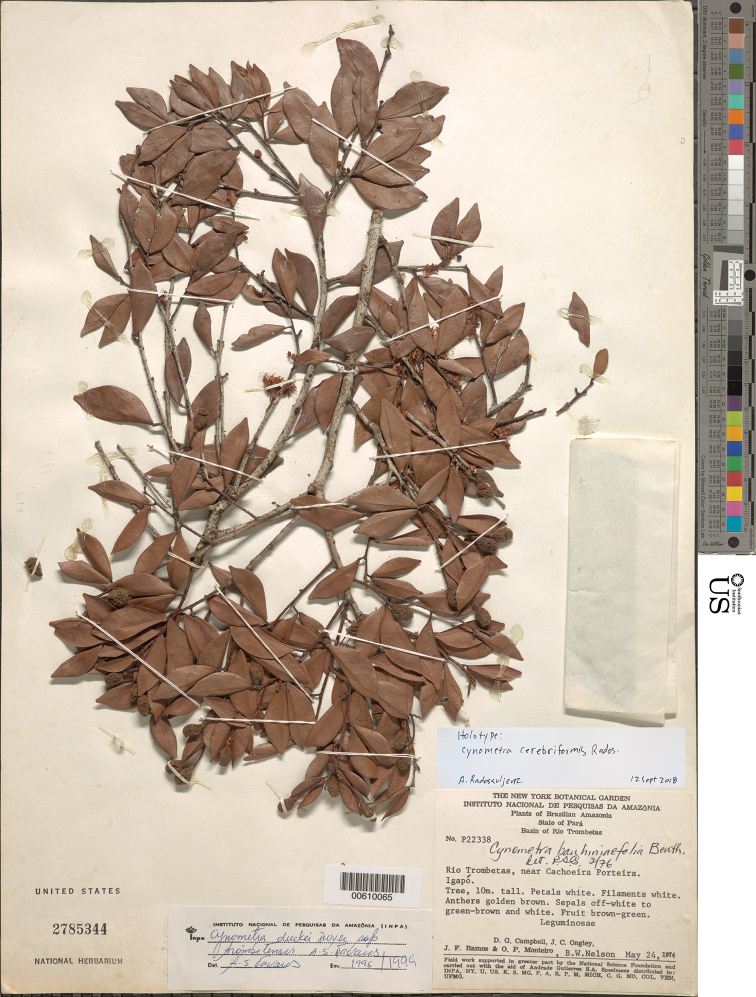
Photograph of the holotype of *Cynometracerebriformis* (*Campbell et al. P22338*, US).

#### Distribution and ecology.

Currently, this species appears restricted to the Trombetas River Basin of Para State, Brazil. However, it is quite possible that the range is more extensive given that the species occurs in seasonally flooded forests and the genus is known for water dispersed fruits (Ridley 1930, Clarke et al. 2001, Tomlinson 2016). Little else is known about this taxon.

#### Phenology.

Flowering specimens have been collected in May. Fruiting specimens have been collected in June and August.

#### Etymology.

*Cynometracerebriformis* is named after the brain-like appearance of the mature fruit.

#### Additional specimens examined.

**BRAZIL. Pará**: Municipio Oriximiná: Rio Trombetas shore, Porteira cemetery, 3 Jun 1974 [im fr], *D. G. Campbell et al. P22510*(INPA, NY, US); Margem direita do Rio Mapuera, entre as Cach[oeira] Paraiso Grande e Maracajá, Área do reservatório da 2^a^ etapa, 00°58'S, 57°35'W, 12 Aug 1986 [im fr], *C.A. Cid Ferreira et al. 7659* (INPA, NY); Rio Trombetas, river banks downriver from Cachoeira Porteira, N to NE bank (Between C.P. and IBDF Reverval – Lago do Jacaré), 17 Jun 1980 [fr], *C. Davidson & G. Martinelli 10324* (INPA, NY, US); Rio Trombetas, margem esq. entre o Lago Jacaré e Cachoeira Porteira, 70 m alt., 17 Jun 1980 [fr], *G. Martinelli 7016* (INPA, NY, RB).

#### Notes.

This taxon has been collected in the areas around Santarem and Oriximiná in Para, Brazil. Many of the specimens have been annotated by Adalea Sprada Tavares as Cynometraduckeissp.trombetensis, but I can find no record of publication and several of the specimens thus annotated are assigned to different taxa in her unpublished thesis (Sprada Tavares 1987). The available material of *C.duckei* is limited and only a few fruiting specimens exist. While a case can be made that *C.duckei* shares certain vegetative traits with *C.cerebriformis* (prominent lenticels, the smoothness of the adaxial surface of the leaflets), it also shares characteristics with several other taxa, including C.spruceanavar.spruceana (long pedicels, leaflet shape) and C.marginatavar.laevis (smooth leaflet surface, nearly sessile leaflets). In light of the characters separating *C.cerebriformis* from other taxa (see below), the author has opted to describe this taxon at the species level.

*Cynometracerebriformis* differs from *C.duckei* primarily in the shape of the leaflets. *Cynometracerebriformis* differs from C.spruceanavar.spruceana in several ways. The leaflets of *C.cerebriformis* are generally smaller than those of C.spruceanavar.spruceana and the surface is nearly smooth, while the secondary veins are quite obvious in *C.spruceana*. *Cynometracerebriformis* also lacks the basal laminar gland present in *C.spruceana* and many other *Cynometra* taxa. Finally, the fruit of *C.spruceana* is approximately 1.5×–2.5× larger than the fruit of *C.cerebriformis* and the valves are smooth to slightly rugulose, lacking the strongly rugose surface of *C.cerebriformis*.

*Cynometracerebriformis* differs from C.marginatavar.laevis by having an acute leaflet base, short acumen and oblate rugose fruit; C.marginatavar.laevis has an obtuse leaflet base, long acumen and a globose fruit with a prominent raised suture ridge.

### 2. Cynometra dwyerii

Taxon classificationAnimaliaFabalesFabaceae

Rados. sp. nov.

urn:lsid:ipni.org:names:77199272-1

Figures 6 , 7

#### Type.

PANAMA. Darién [now Comarca Emberá-Wounaan]: vicinity of Campamento Buena Vista, Río Chucunaque above confluence with Río Tuquesa, [08°23'N, 77°47'W] 5 July 1959, [fr.], *W. L. Stern 941* (holotype US; isotype MO).

#### Description.

***Tree*** to approximately 20 m tall; bark not seen; branchlets lenticelate, pubescent when young, becoming glabrous with age. ***Stipules*** not seen. ***Leaves*** bifoliolate, axes pubescent, transversely corrugated; petioles 4.5–5.5 mm long; petiolules 1.0–1.5 mm long, inconspicuous, leaflets appearing sessile; leaflets narrowly obovate to obovate, occasionally sub-trapeziform, strongly asymmetric, primary vein eccentric, proximal side 2.8–3.7 times wider than distal, 3.1–3.9 cm long, 1.4–1.9 cm wide, thin, abaxial surface sparsely pubescent, more so on midvein and major secondaries, adaxial surface with pubescence restricted primarily to midvein, occasional hairs scattered on lamina, primary venation pinnate, secondary venation brochidodromous-eucamptodromous, 2(–3) basal acrodromous veins, decurrent to primary, prominent abaxially, only slightly less so adaxially, tertiary venation visible on both surfaces at 10× magnification, margins entire, apex acute, weakly acuminate (to 2.0 mm), retuse, mucronate, base oblique, acute, distal side narrowly cuneate, proximal side slightly concave to cuneate, decurrent to petiolule, laminar glands present, 3–6 per leaflet, arranged in a row approximately halfway between margin and midvein, restricted to distal portion of lamina, typically adjacent to tertiary veins, crateriform, less than 1.0 mm in diameter. ***Inflorescences*** not seen, position inferred as axillary from remnant of peduncle. ***Flowers*** not seen. ***Legume*** indehiscent, roughly globose, to 4.7 cm in diameter, surface of valves rugulose, wall of pericarp up to 3.0 mm thick, deep brown colour at maturity. ***Seeds*** 1 per pod, filling entire cavity, dark brown.

**Figure 6.**
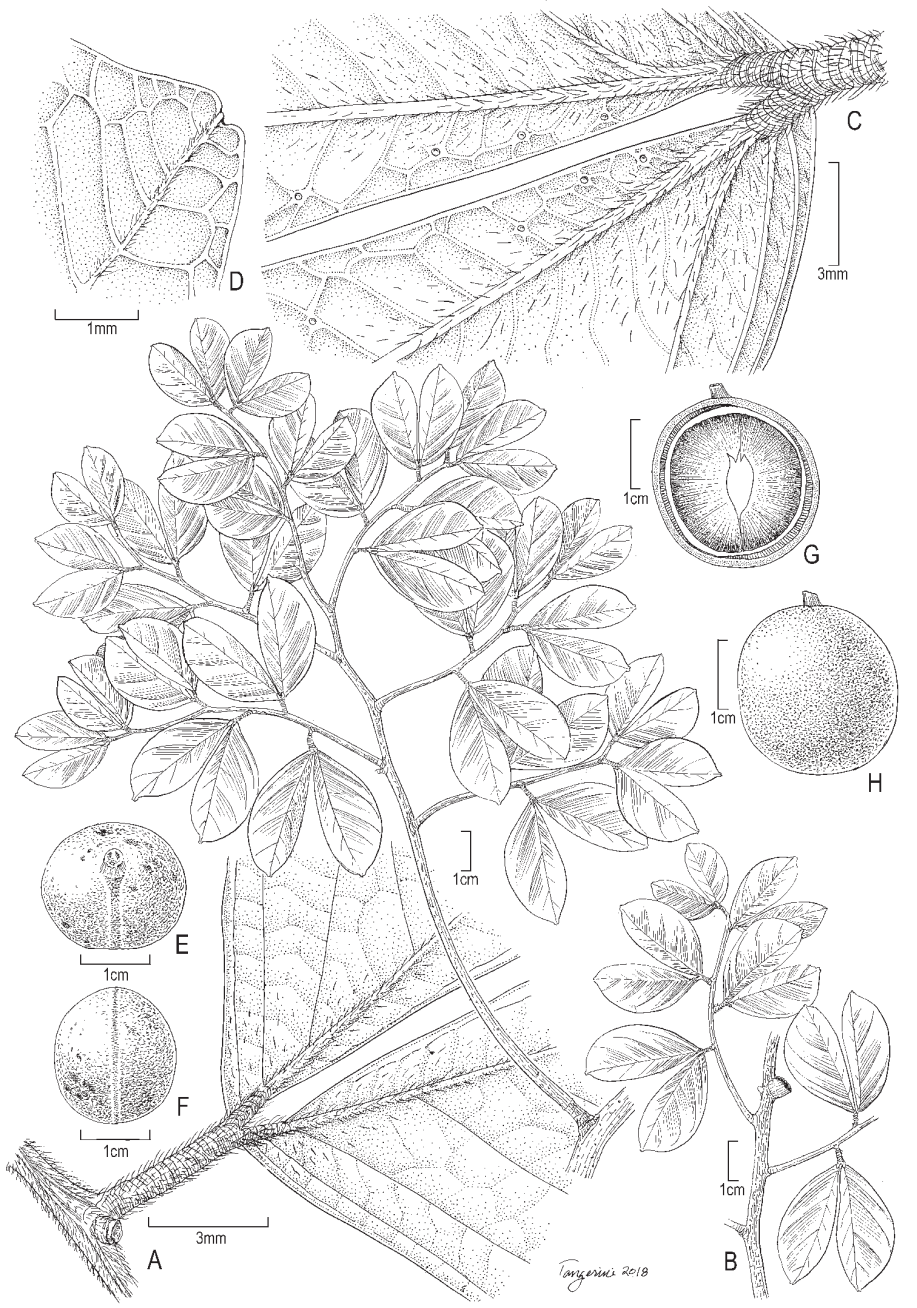
Illustration of *Cynometradwyerii*. **A** Leaf base (adaxial surface) showing corrugated petiole and pubescence along midrib **B** habit **C** leaf base (abaxial surface) showing basal acrodromous veins arising from leaflet pulvinus and laminar glands **D** leaflet apex (abaxial surface) **E** Dissected fruit, proximal surface **F** dissected fruit, distal surface **G** longitudinal section of fruit with single seed **H** reconstruction of fruit. **A–H***Stern et al. 941*, US.

**Figure 7.**
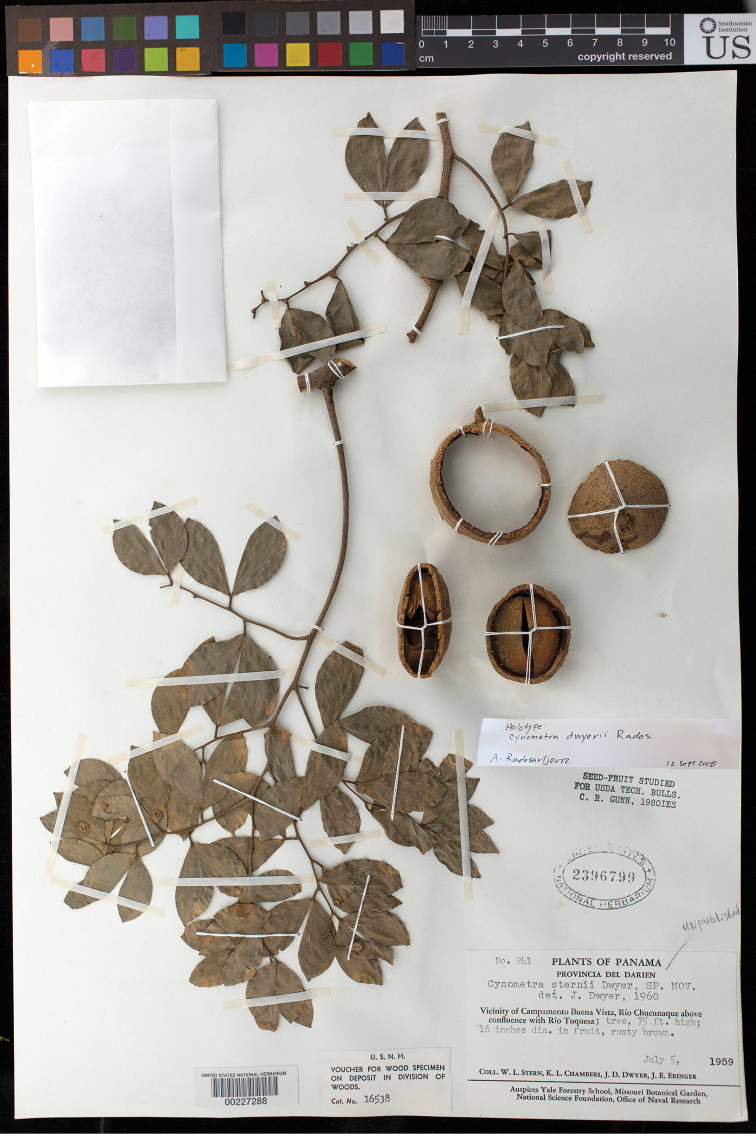
Photograph of the holotype of *Cynometradwyerii* (*Stern et al. 941*, US).

#### Distribution and ecology.

Known only from the type locality in the Darien Gap region of Panama; the area where the type was collected is primarily lowland moist tropical forest.

#### Phenology.

The type was collected with mature fruit in July.

#### Etymology.

The specific epithet honours the contributions of Dr. John Dwyer, who published the first monograph of the Neotropical species of *Cynometra*.

#### Note.

This species is known only from the type collection, however the combination of vegetative characters and fruit morphology make it clearly distinct from other *Cynometra* species. Superficially, this species resembles *C.bauhiniifolia*, given its small leaflets with prominent secondary venation. However, the pubescence and arrangement of laminar glands clearly distinguish it from other Neotropical species of *Cynometra*, which usually have just a single, basal laminar gland. In fact, the combination of bifoliolate leaves with small leaflets and several submarginal laminar glands is unique across the entire genus. When these characters are combined with the large, globose fruit, it is clear that this is a distinct species.

#### Additional specimens examined.

None.

### 3. Cynometra steyermarkii

Taxon classificationAnimaliaFabalesFabaceae

Rados. sp. nov.

urn:lsid:ipni.org:names:77199276-1

Figures 8 , 9 , 10

#### Type.

VENEZUELA. Miranda: Distrito Brión, Selva siempre verde a lo largo de la quebrada afluente del río Aricagua, 3.9 km oeste del Pueblo Seco, 1.6 km oeste de Aricagua, 75 m alt., 24–25 March 1973, [fl, fr], *J. A. Steyermark & V. Carreño Espinoza*, *106937* (holotype: US; isotypes: F, VEN *n.v*.).

#### Description.

***Tree*** to 25 m tall; bark not seen, sapwood reddish; branchlets glabrous, lenticelate. ***Stipules*** not seen. ***Leaves*** bifoliolate, axes glabrous; petioles 6.5–9.0 mm long, 2.0 mm wide, transversely corrugated; petiolules 2.0–3.0 mm long, 1.0 mm wide, transversely corrugated; leaflets, coriaceous, elliptic to slightly obovate, asymmetric, primary vein eccentric, proximal side 2.3–3.1 times wider than distal, 5.7–7.1 cm long, 2.7–3.6 cm wide, discolorous, abaxial and adaxial surface glabrous, primary venation pinnate, secondary venation brochidodromous-eucamptodromous, 2(–3) basal acrodromous veins, decurrent to primary vein, prominent abaxially, slightly raised adaxially, tertiary venation visible on abaxial surface without magnification, margins entire, apex obtuse, usually rounded but occasionally acuminate (to 3.0 mm), retuse, mucronate, base oblique, acute, distal side strongly cuneate, proximal concave to convex, decurrent to petiolule, single laminar gland present on some leaflets, abaxial, near basal margin of proximal lamina and insertion point of petiolule, typically adjacent to tertiary veins, crateriform, 0.5 mm in diameter. ***Inflorescence*** an axillary raceme, bracteate, axes densely ferrugino-puberulent; peduncle together with rachis to 6.0 mm long, flowers spirally arranged, 15 per raceme; pedicels 5.5–6.0 mm, pubescent, accrescent in fruit; bracts subtending individual flowers, scale-like, deciduous, lustrous, brown, broadly elliptical, strongly convex 1.0–2.0 mm long, 1.5–2.5 mm wide, striate, abaxial surface with appressed pubescence along medial surface and along margins, glabrous adaxially; bracteoles paired, inserted near base of pedicel, subopposite, linear to oblanceolate, 2.5–3.0 mm long, 0.75 mm wide, acute at apex, densely pubescent on abaxial surface, less so adaxially. ***Flowers*** bisexual, radially symmetric, pentamerous, delicate; hypanthium much reduced, less than 0.5 mm deep, with a few scattered hairs on abaxial and adaxial surface; sepals 4, imbricate, reflexed, slightly unequal, white, petaloid, oblong to obovate, 2.5–3.5 mm long, 1.0–1.5 mm wide, faintly striate, appressed pubescence on abaxial surface near insertion point and along central axis; petals 5, equal, white, oblanceolate, 3.5–4.5 mm long, 1.0–1.5 mm wide, pinnate venation; stamens 10, filaments appearing free but possibly connate for approximately 0.25 mm at base, subequal, 4.0–5.5 mm long, anthers dorsifixed, versatile, longitudinal dehiscence, ellipsoid, to 0.75 mm long, glabrous; ovary centrally inserted, free, sessile, obliquely elliptical, 2.5–4.0 mm long, 1.5–3.0 mm wide, pubescent at base and along suture, becoming glabrous with age, style apical, 1.5–2.5 mm long, glabrous, eccentric, stigma capitate. ***Legume*** indehiscent, globose, shortly apiculate (to 0.75 mm), 1.5 cm in diameter, surface of granulose, with very short pubescence, wall of pericarp up to 2.0 mm thick, brown. ***Seeds*** 1 per pod (1 ovule per ovary based on dissections), dark brown.

**Figure 8.**
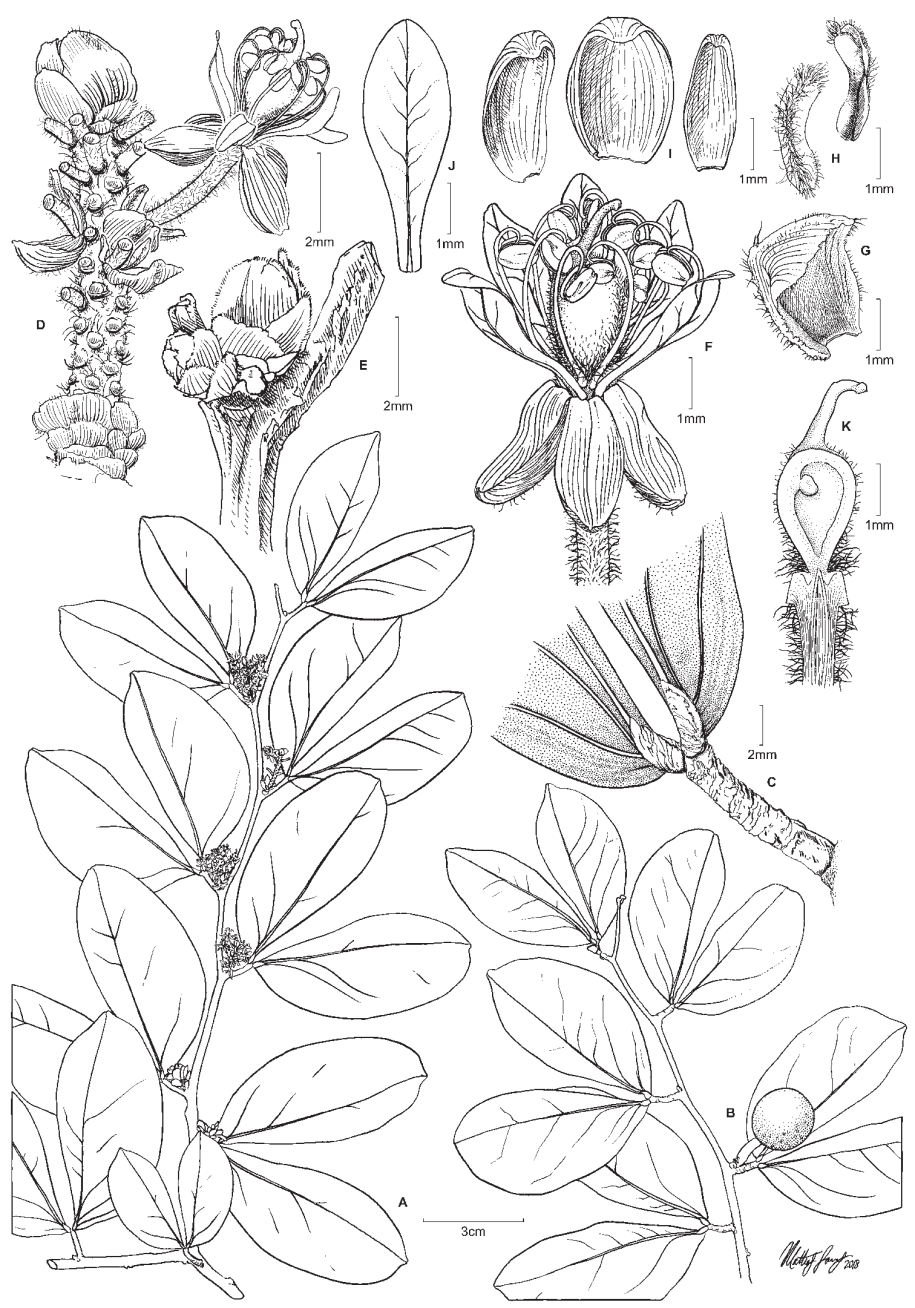
Illustration of *Cynometrasteyermarkii*. **A** Habit, flowering branch **B** habit, fruiting branch **C** leaf base, showing basal acrodromous veins arising from leaflet pulvinus **D** inflorescence rachis, showing bracts and bracteoles; pedicels removed to show structure **E** buds with imbricate bracts **F** flower **G** bract **H** bracteoles **I** sepals **J** petal **K** longitudinal section of flower showing single ovule and much reduced hypanthium; sepals, petals and stamens removed. **A–K***Steyermark et al. 106937*, US.

**Figure 9.**
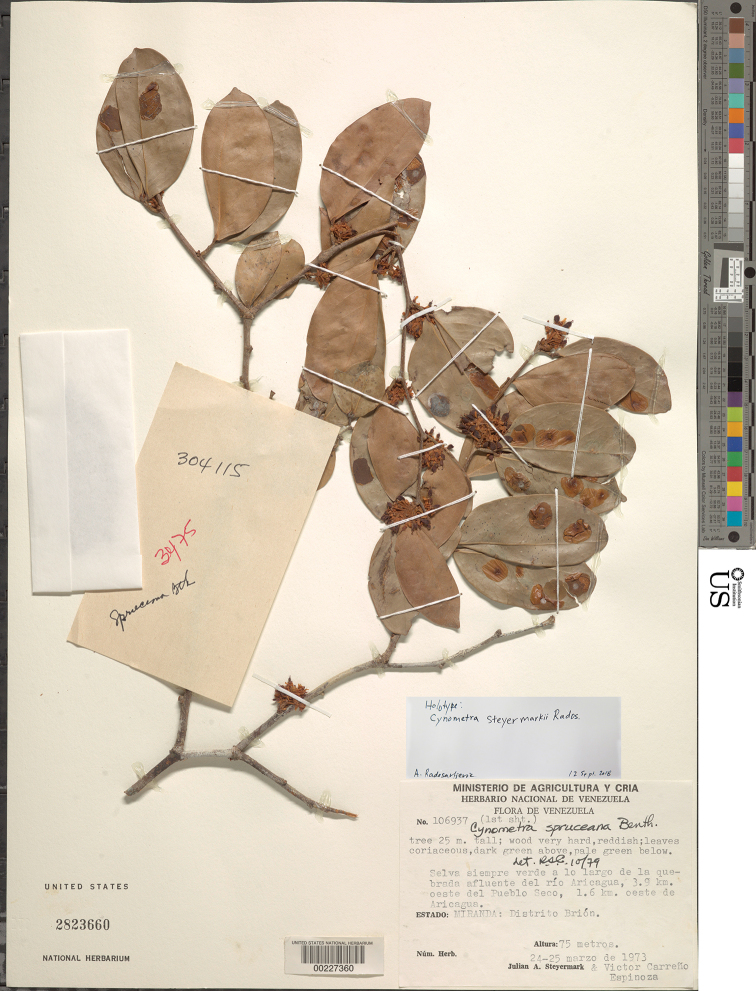
Photograph of the holotype of *Cynometrasteyermarkii*, sheet one of two (*Steyermark et al. 106937*, US).

**Figure 10.**
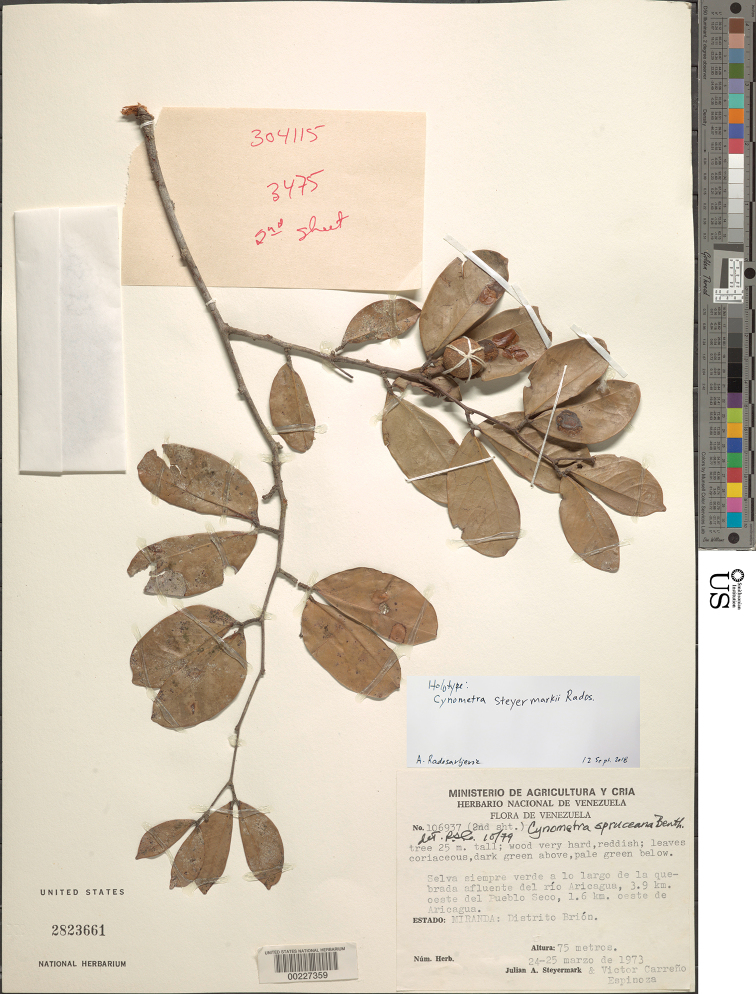
Photograph of the holotype of *Cynometrasteyermarkii*, sheet two of two (*Steyermark et al. 106937*, US).

#### Distribution and ecology.

Known from a single collection made in Miranda State, Venezuela. The species was collected growing along a small stream in the foothills of the Cordillera de la Costa at approximately 75 m above sea level. While much of this region is covered in xeric shrublands and thorn forests, this site corresponds to a low gallery forest growing along a tributary of the Río Aricagua.

#### Phenology.

The type material was collected at the end of March with late flowers and nearly mature fruit.

#### Etymology.

The specific epithet honours Dr. Julian A. Steyermark, the collector of the type material and a prolific collector of neotropical plants. Over the course of his career, he made over 130,000 collections and greatly advanced our knowledge of the Venezuelan flora.

#### Additional specimens examined.

None.

#### Notes.

*Cynometrasteyermarkii* is the only confirmed species of *Cynometra* known from the forests of the Cordillera de la Costa in northern Venezuela and one of two species of Venezuelan *Cynometra* growing outside of the Guayana region. Henri Pittier (1926) published an account of a *Cynometra* growing in the hills outside of Caracas, *Cynometrasphaerocarpa* Pittier, for his *Manual de las plantas usuales de Venezuela.* While the description is valid, according to the rules of nomenclature in effect at the time, he failed to designate a type or list any collections that may correspond to this taxon. In his 1958 revision, Dwyer was unable to locate any material that could be assigned to this taxon and pointed out the limited utility of Pittier’s description – ‘Las hojas inequilaterales, lanceadas, largamente atenuadas y glabras… Los frutos son subglobosos, de 3.5–4 cm. de diametro y contienen una sola semilla [Leaflets asymmetric, lanceolate, largely attenuate and glabrous. The fruits are sub-globose, 3.5–4 cm in diameter, and contain a single seed].’– which could very well describe any number of *Cynometra* species, including *C.steyermarkii*. A full set of Pittier’s duplicates from this publication should be deposited in the United States National Herbarium (US), but after exhaustive searches in the US collections and a study of both Pittier’s archives and the museum registrar’s records, I was unable to find any material that could be attributable to *C.sphaerocarpa*. Curators at Herbario Nacional de Venezuela graciously searched through material housed there, but were also unsuccessful. While it is possible that *C.steyermarkii* and *C.sphaerocarpa* are the same taxon, without Pittier’s original material, it is impossible to know for sure. Rather than leave this unresolved, it is more useful to describe a new species with good type material.

This species bears a resemblance to Cynometraspruceanavar.phaselocarpa Benth. owing to the obtuse nature of the leaf apices. However, *C.steyermarkii* often has slightly acuminate apices. Furthermore, both the rachis and pedicels of the inflorescence are much shorter in *C.steyermarkii* than in typical C.spruceanavar.phaselocarpa.

### 4. Cynometra tumbesiana

Taxon classificationAnimaliaFabalesFabaceae

Rados. sp. nov.

urn:lsid:ipni.org:names:77199277-1

Figures 11 , 12

#### Type.

ECUADOR. El Oro: Bosque Petrificado Puyango, dirt track from information centre towards the camping area near Río Puyango, 03°52'30"S, 80°05'01"W, 450 m alt., 6 May 1997 [fl], *B.B.Klitgaard et al. 507*, (holotype K; isotype AAU n.v., LOJA n.v., NY, QCNE n.v., US).

#### Description.

***Tree*** 10–25 m tall; bark grey-brown, lenticelate, inner bark red; branchlets with short pubescence when young, becoming glabrous with age. ***Stipules*** not seen. ***Leaves*** bifoliolate, axes ferrugino-puberulent when young, glabrous when mature; petioles 4.5–6.5 mm long, transversely corrugated; petiolules 1.5–2.0 mm long, inconspicuous; leaflets oblong-ovate to elliptic to oblong-obovate, occasionally slightly falcate or sub-trapeziform, strongly asymmetric, primary vein eccentric, proximal side 1.8–2.5 times wider than distal, 4.1–7.9 cm long, 2.5–3.3 cm wide, discolorous, abaxial surface sparsely pubescent on midvein, secondary veins and along basal margin, adaxial surface glabrous, primary venation pinnate, secondary venation brochidodromous-eucamptodromous, 2–3 (–4) basal acrodromous veins, decurrent to primary vein, prominent abaxially, flush to slightly raised adaxially, tertiary venation visible on abaxial surface at 10× magnification, margins entire, apex acute, acuminate (to 6.0 mm), retuse, mucronate, base oblique, distal side acute, convex to cuneate, proximal side obtuse, concave to rounded, decurrent to petiolule, single laminar gland present, abaxial, near basal margin of proximal lamina and insertion point of petiolule, typically adjacent to tertiary veins, crateriform, 1.0 mm in diameter. ***Inflorescence*** a cluster of (1–)2–3 axillary racemes, bracteate, axes ferrugino-puberulent at base, hairs becoming scattered at distal end; peduncle together with rachis 4.5–8.0 mm long, flowers spirally arranged, 12–20 per raceme; pedicels 5.0–9.0 mm, pubescent initially but soon glabrescent, accrescent in fruit; bracts subtending individual flowers, scale-like, deciduous, leaving behind a lunate scar on the rachis, lustrous, brown, broadly elliptical, strongly convex 1.5–2.5 mm long, 1.5–2.5 mm wide, striate, abaxial surface with scattered appressed pubescence at apex and along margins, glabrous adaxially; paired bracteoles inserted 0.5–1.0 mm from base of pedicel, subopposite, oblong-lanceolate, 2.0–2.5 mm long, 1.0 mm wide, convex at apex, pubescent along margins and medial abaxial surface. ***Flowers*** bisexual, radially symmetric, pentamerous, delicate; hypanthium cupular, 1.0–1.5 mm deep, fleshy, with a few scattered hairs on abaxial and adaxial surface; sepals 4, imbricate, reflexed, unequal, adaxial sepal usually 2 times as wide as the others, white, petaloid, broadly ovate to elliptical, 3.0–4.5 mm long, 1.5–4.0 mm wide, striate, scattered pubescence at base; petals 5, erect, equal to subequal, white, spathulate to oblanceolate, 3.5–5.5 mm long, 1.0–2.0 mm wide, glabrous but with a tuft of hair at base of claw; stamens 10, filaments free, subequal, 5.5–7.5 mm long, white, anthers dorsifixed, versatile, longitudinal dehiscence, suborbicular, to 1.5 mm long, yellow-orange, glabrous; ovary centrally inserted in hypanthium, free, stipitate, obliquely elliptical, 4.0–5.0 mm long, 2.0–2.5 mm wide, densely pilose, stipe 0.5–1.0 mm long, style apical, 3.0–4.0 mm long, glabrous, eccentric, geniculate, stigma capitate. ***Legume*** indehiscent, oblong, weakly apiculate, slightly compressed, up to 5.2 cm long, 4.0 cm wide, 3.9 cm thick, surface of valves finely textured, granulose, wall of pericarp up to 4.0 mm thick, deep brown colour at maturity. ***Seeds*** 1 per pod, filling entire cavity, dark brown.

**Figure 11.**
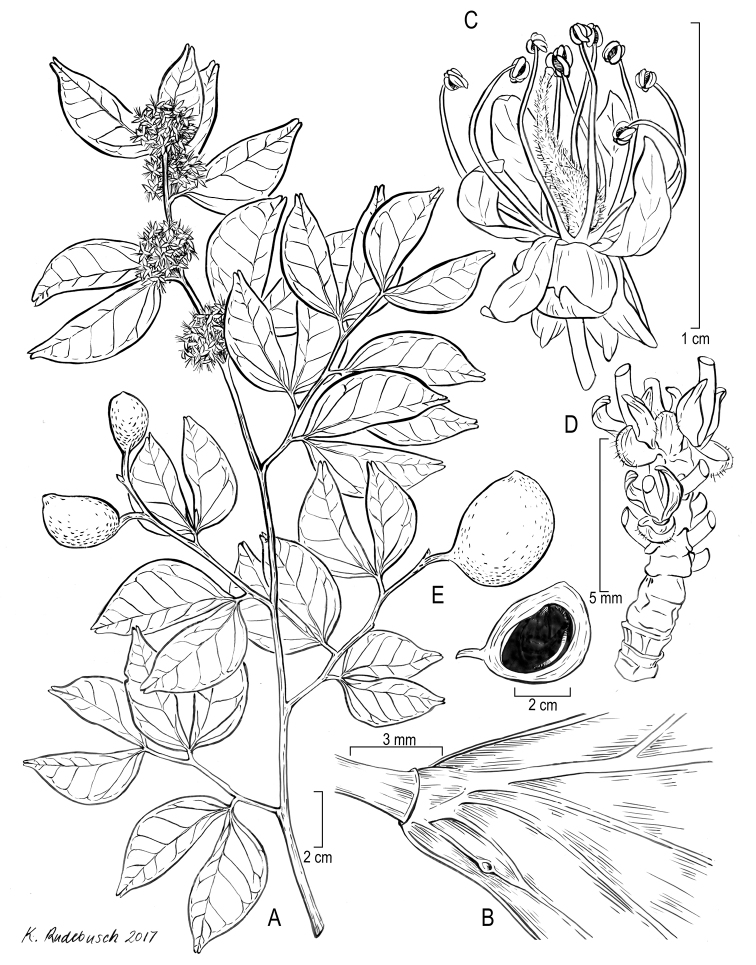
Illustration of *Cynometratumbesiana*. **A** Habit **B** leaflet base, showing laminar gland **C** flower **D** inflorescence rachis, showing bracts and bracteoles; pedicels removed to show structure **E** fruit, dissected to show single seed filling entire cavity. **A–D***Klitgaard et al. 507*, K; **B***Neill & Núñez 10453*, US.

**Figure 12.**
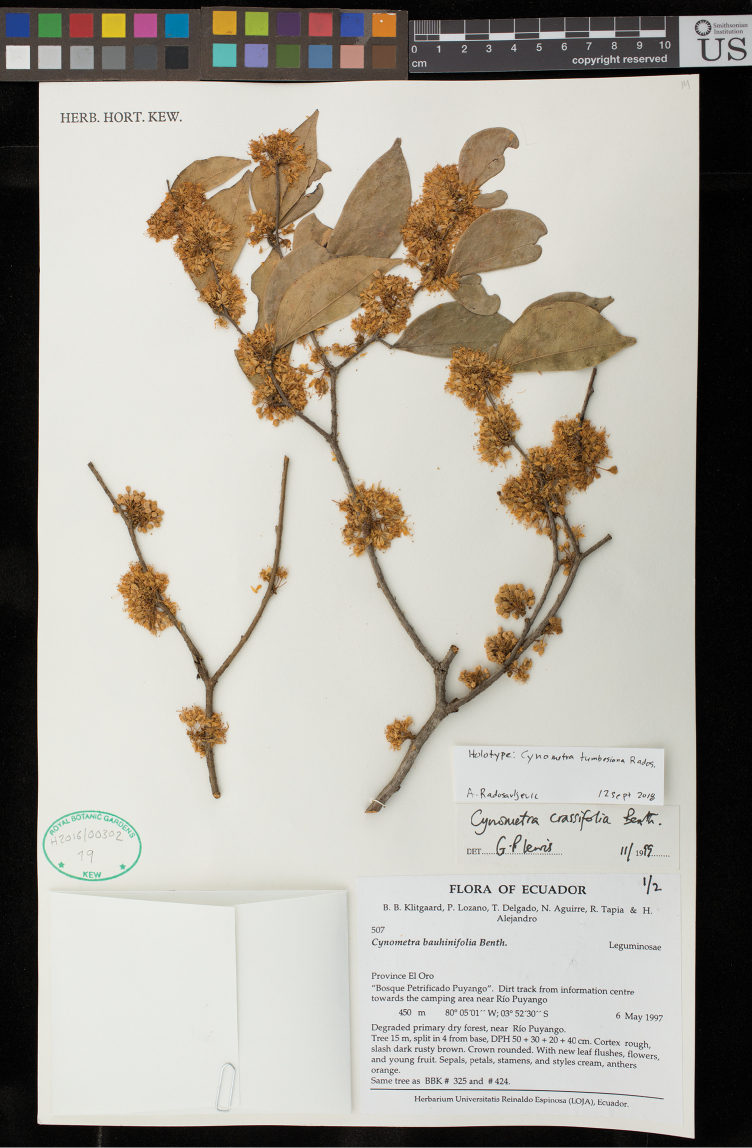
Photograph of the holotype of *Cynometratumbesiana* (*Klitgaard et al. 507*, K).

#### Distribution and ecology.

*Cynometratumbesiana* occurs in the seasonally dry tropical forests of western and southern Ecuador and north-western Peru (a single collection) at elevations between 100–800 m (Figure 3). These habitats are severely threatened regionally and globally due to extensive human modification of the landscape and, as a result, this species now exists primarily as isolated fragments. While *C.tumbesiana* is locally abundant at a few sites, it is currently known from less than 10 localities, several of which are within 5 km of each other.

#### Phenology.

Flowering specimens have been collected in May and December to January; fruiting specimens have been collected in August and January to February. *Cynometratumbesiana* is one of the few woody taxa in the dry forests that retain their leaves during the dry season.

#### Etymology.

The specific epithet refers to the Tumbes region, where the type specimen was collected and where many of the known localities occur.

#### Additional specimens examined.

**ECUADOR. El Oro**: Bosque Petrificado Puyango, dirt track from information centre towards the camping area near Río Puyango, 03°52'30"S, 80°05'01"W, 450 m alt., 23 Aug 1996 [fr], *B.B. Klitgaard et al. 325* (AAU n.v., K, LOJA n.v., NY, QCNE n.v.); 26 Feb 1997 [fr], *B.B. Klitgaard et al. 424* (AAU n.v., K, LOJA n.v., NY, QCNE n.v.). [Piñas]: Piedras, about 3 km. along new trail, 18 Jun 1943 [st], *E.L. Little*, *Jr. 6622* (US). **Guayas**: [without specific locality] 2 Feb 1962 [fr], *A.J. Gilmartin 551* (US). Guayaquil: Bosque Protector Cerro Blanco, 15 km west of Guayaquil, summit area of Cerro Blanco, 2°10'S, 79°58'W, 370 m alt., 27 Feb 1996 [fr], *D. Neill & T. Núñez 10453* (MO, US); Bosque Protector Cerro Blanco, along road from visitor centre to “Cusumbo Top”, 80, 01 W, 2 10 S, 400 m alt., 7 Aug 1996 [im fr], *D. Neill*, *T. Núñez & J. Machuca 10636* (MO); Bosque Protector Cerro Blanco, carretera a Salinas, km 15, 2°10'S, 79°58'W, 400 m alt., 21–25 Jan 1992 [fr], *D. Rubio & Galo Tipaz 2365* (MO). Isidro Ayora: Reserva Ecológica Manglares Churute, carretera Guayaquil–Puerto Inca, sector norte del Cerro Masvale, 2°20'S, 79°50'W, 200–300 m alt., May 1993 [fl], *T. Núñez & A. Hernández 147* (MO). **Manabi**: [Puerto López]: Estero Perro Muerto, Machalilla National Park, below San Sebastian, 1°36'S, 80°42'W, 400–420 m alt., 23 Jan 1991 [fl], *A. Gentry & C. Josse* 72677 (MO); [San Vicente]: [hacienda] El Recreo, [fl], *H.F.A. von Eggers 15752* (US). **PERU. Tumbes**: Zarumilla: Dtto. Matapalo, Campo Verde a 68 km de. Tumbes, 700–800 m alt., 24 Dec 1967 [fr], J. Schunke V. 2411 (F, NY, US).

#### Notes.

This species, restricted to the few remaining fragments of dry tropical forest in western Ecuador and the Tumbes region of Peru, has been mistakenly referred to as *Cynometracrassifolia* Benth. for many years. However, closer examination shows it to be quite distinct from this taxon. The type specimen of *C.crassifolia* was collected in Brazil by Portuguese naturalist Alexandre Rodriques Ferreira during his exploration of the Amazonian region of Brazil from 1783–1792. His collections, along with many others housed at Lisbon, were expropriated by Étienne Geoffroy Saint-Hilaire and transferred to Paris during Napoleon’s occupation of Portugal. There, it was seen by George Bentham, who described *Cynometracrassifolia* in 1840. The primary differences between *C.tumbesiana* and *C.crassifolia* are found in the inflorescences: the racemes of *C.crassifolia* have larger flowers, longer pedicels and a more robust pedicel and rachis, but fewer individual flowers than those of *C.tumbesiana*. The flowers of *C.tumbesiana* are indeed relatively small compared to the other neotropical *Cynometra* species, though they are densely clustered on the short rachis of the inflorescence. The leaflets of *C.tumbesiana* are also less distinctly acuminate than those of *C.crassifolia* and have a less obtuse base; the base of the leaflet in *C.crassifolia* can appear to be almost truncate.

Some taxonomists have placed the Ecuadoran *Cynometra* within *C.bauhiniifolia* Benth., with a few treating *C.crassifolia* as a synonym of *C.bauhiniifolia* (Neill et al. 1999). In the first case, while *C.tumbesiana* does bear a passing resemblance to some forms of *C.bauhiniifolia*, the pod of *C.bauhiniifolia* is many times smaller and the surface of the valves is corky and deeply rugose. In the latter case, it is difficult to find justification for synonymising the two with the exception that *C.bauhiniifolia* has been a dumping ground for hard to place taxa within the genus and does occasionally occur in drier habitats. The leaflets, inflorescences and fruits all differ. Instead, the type material of *C.crassifolia* seems to be very similar to *C.longicuspis* Ducke, a widespread species from the moist lowland forests of Brazil.

*Cynometratumbesiana* is morphologically and ecologically similar to *C.oaxacana* Brandegee from western and southern Mexico. The two can be distinguished by the narrower and more acuminate leaflets and larger fruits in *C.tumbesiana*. The inflorescences of *C.oaxacana* are also slightly more robust and have a more obvious pubescence. Both species are found in dry habitats (uncommon amongst the neotropical species of *Cynometra*), though *C.tumbesiana* is found in much drier sites.

## Supplementary Material

XML Treatment for Cynometra basifoliola

XML Treatment for Cynometra brassii

XML Treatment for Cynometra browneoides

XML Treatment for Cynometra cynometroides

XML Treatment for Cynometra fortuna-tironis

XML Treatment for Cynometra lenticellata

XML Treatment for Cynometra lenticellata var. villosa

XML Treatment for Cynometra mariettae

XML Treatment for Cynometra megalocephala

XML Treatment for Cynometra minor

XML Treatment for Cynometra plurijuga

XML Treatment for Cynometra psilogyne

XML Treatment for Cynometra rosea

XML Treatment for Cynometra schefferi

XML Treatment for Cynometra schefferi var. peekelii

XML Treatment for Cynometra steenisii

XML Treatment for Cynometra steenisii var. rodneyensis

XML Treatment for Cynometra vestita

XML Treatment for Cynometra vitiensis

XML Treatment for Cynometra cerebriformis

XML Treatment for Cynometra dwyerii

XML Treatment for Cynometra steyermarkii

XML Treatment for Cynometra tumbesiana
